# Parasites’ immunomodulators: a breakthrough in immunotherapeutics displaying antineoplastic activity against human colorectal and hepatocellular carcinoma cells

**DOI:** 10.1186/s13027-025-00715-6

**Published:** 2025-12-12

**Authors:** Nahla El Skhawy, Ahmed Shehata, Maha M. Eissa

**Affiliations:** 1 Department of Medical Parasitology, Faculty of Medicine, Alexandria University, Alexandria, Egypt; 2 Joint MB ChB/ MBBCH Programme, Faculty of Medicine, Alexandria University-Manchester University, Alexandria, Egypt

**Keywords:** Immunomodulators, Parasitic antigens, Parasitic antibodies, Colorectal carcinoma, Hepatocellular carcinoma

## Abstract

The complex interplay between parasites and cancer is yielding promising advances in the field of cancer therapeutics. This study explored the in vitro anti-cancer potential of parasite immunomodulators (antigens and antibodies) of *Schistosoma mansoni*,* Trichinella spiralis*, and *Toxoplasma gondii* on human HT-29 colorectal and HepG2 hepatocellular carcinoma cells using the MTT assay. Results revealed that those parasites’ immunomodulators exhibited antineoplastic activity and demonstrated a statistically significant inhibition of both cancer cell lines’ proliferation (P ˂0.05). Notably, *Trichinella spiralis* antigens and antibodies and anti-*Toxoplasma gondii* antibodies demonstrated the most statistically significant inhibitory effects on HT-29 colorectal cancer cells (36.65%, 49.9% and 50.43% respectively). For HepG2 hepatocellular carcinoma cells, *Trichinella spiralis* antigens and antibodies, as well as *Toxoplasma gondii* antigen, displayed the most statistically significant inhibitory effects (38.27%, 48.25% and 34.68% respectively). Interestingly, parasitic antibodies are particularly noteworthy, exhibiting the most significant inhibitory effects on both cancer cell lines. To the best of our knowledge, this is the first study to demonstrate the antineoplastic activity of parasites’ antibodies against human colorectal cancer and hepatocellular carcinoma. These findings could enlighten the path for promising cancer therapeutic candidates. Future research should explore the antineoplastic potential of a broader range of parasites’ immunomodulators and identify their mechanisms of action. This could represent a qualitative shift towards the development of innovative cancer therapeutic antibodies and cancer vaccine candidates of parasitic origin for cancer-targeted immunotherapy.

**Graphical Abstract:**

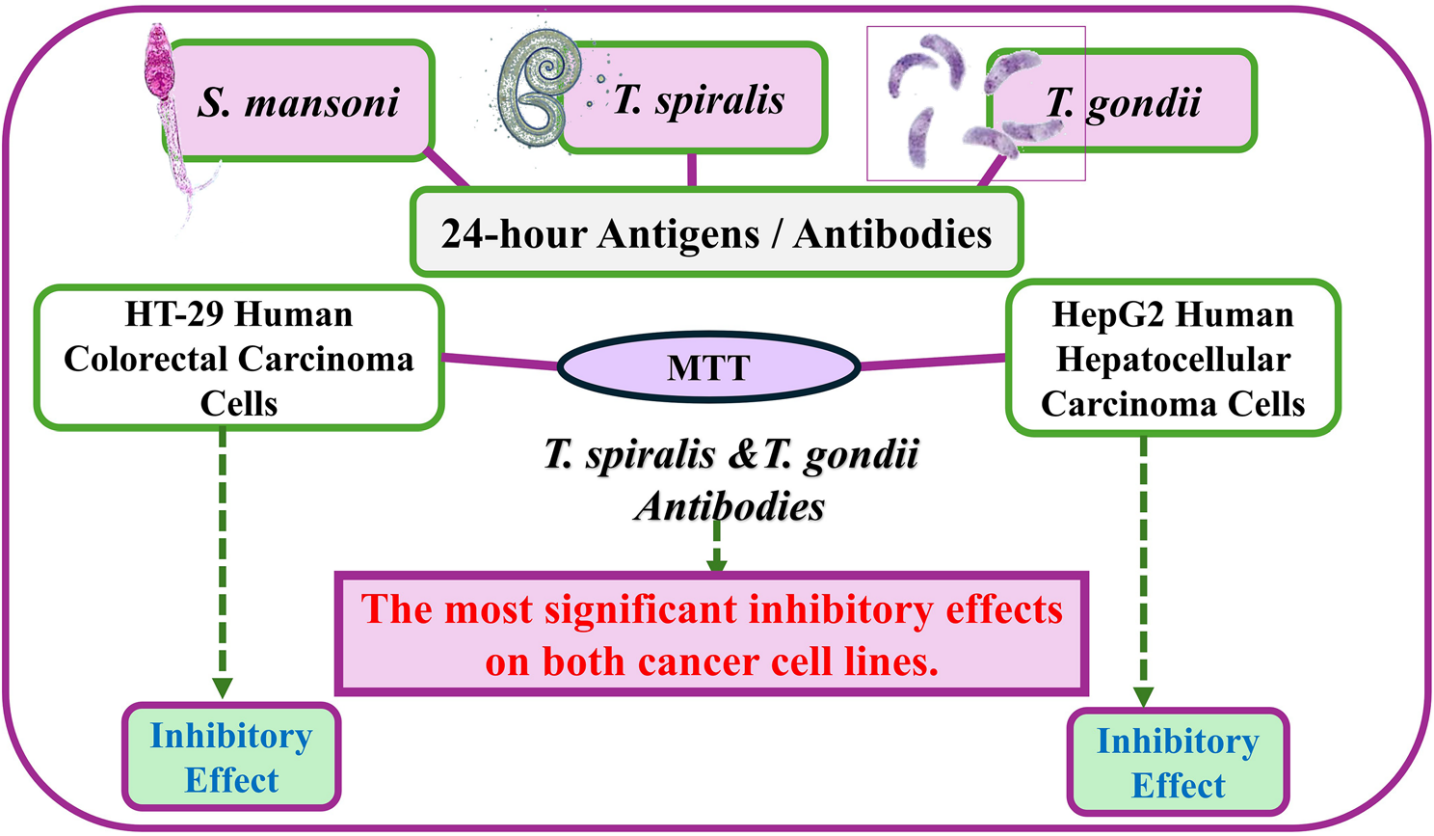

**Supplementary Information:**

The online version contains supplementary material available at 10.1186/s13027-025-00715-6.

## Introduction

Cancer is a global health concern with a dramatic influence on patients’ quality of life, productivity, and psychological aspects [1]. Gastrointestinal cancers (GICs) constitute a huge bulk of cancer cases, of which colorectal carcinoma (CRC) is the greatest burden. Gastrointestinal cancers account for 26% of cancer incidences (5.26 million cases) and 35% of cancer deaths (3.70 million fatalities) globally in 2021 [2, 3], within which liver cancer, mainly hepatocellular carcinoma (HCC), is considered the third most common cause of cancer-related deaths globally [4]. While chemotherapeutics, radiotherapy, and surgery are the cornerstones of GICs’ treatment, significant drawbacks exist that hinder their full safe clinical applicability. Thus, immunotherapy has emerged, aiming to enhance the suppressed immune response [5]. In parallel, researchers are seeking alternative cancer candidates that could be more immunogenic, safe, and have homology to cancer antigens.

Long ago, researchers were skeptical regarding the relationship between parasites and cancer due to the association of some parasites with carcinogenesis as *Clonorchis sinensis*, *Schistosoma haematobium*, and *Opisthorchis viverrini* [6]. Nevertheless, other parasites have recently been certified experimentally as anti-cancerous agents via multiple mechanistic strategies [7, 8]. This may be related to the hygiene theory, which postulates that the lack of exposure to pathogenic agents, such as parasites, may be related to an increased prevalence of immune-mediated illnesses and cancer [9]. Additionally, retrospective studies have demonstrated that the prevalence of cancer is notably lower in patients with parasitic diseases such as hydatid or Chagas’ disease compared to the general population [10, 11]. Subsequently, the antineoplastic activities of some parasites were verified experimentally against different cancer models [7, 12].

The cytotoxic effects of certain parasites and their derivatives on cancer cells may be valuable tools towards attacking cancer cells either directly or by manipulating molecular processes at the proteomic and genetic levels to induce cell apoptosis [7, 8]. Although live and attenuated *T. gondii* were able to directly invade and replicate within cancer cells and lead to their lysis, safety concerns may restrict their clinical applicability [13–19]. Thus, using live parasites to promote anti-cancerous effects sounds unreasonable and impractical; consequently, parasite immunomodulators (PIMs) (antigens and antibodies) may hold an optimistic value, as they are highly immunogenic and display homology to cancer antigens [7].

Interestingly, certain parasitic antigens exhibited direct cytotoxic effects on cancer cells, as antigens from *Echinococcus granulosus (E. granulosus)* [20] and *Toxoplasma. gondii (T. gondii)* [21]. In particular, autoclaved parasitic antigens exhibited potent immunogenicity against their corresponding parasitic infections, as experimental schistosomiasis [22], trichinellosis [23], toxoplasmosis [24], among others [25, 26]. Further data supported their safety [27–29] and their immunomodulatory efficacy against non-parasitic diseases as experimental arthritis [30], psoriasis [31], and cancer models [28, 29, 32, 33]. Thus, investigating the potency of those antigens on human cancer cell lines in vitro deserves further investigation.

Therapeutic monoclonal antibodies have gained tremendous value as supplemental cancer immunotherapeutic agents [34]. Mechanistically, these antibodies can selectively target and destroy cancer cells while sparing healthy cells. These antibodies can activate the complement pathway, stimulate the direct antibody-dependent cell cytotoxicity, induce signalling perturbation, interfere with growth factor receptors, restrict tumor cell proliferation, and opsonize cancer cells, consequently flagging the tumor cells and inviting immune effector cells [34]. However, the role of parasitic antibodies on CRC and HCC cells hasn’t been investigated in depth.

Based on the aforementioned data and the highlighted areas of research gaps between parasites and cancer, we aimed to experimentally screen *Schistosoma mansoni* (*S. mansoni*), *Trichinella spiralis* (*T. spiralis*) and *T. gondii* immunomodulators against two human cancer cell lines: the HCT-29 human CRC and the HepG2 human HCC cell lines.

## Materials and methods

### Ethics statement

All experimental mice were handled following the ARRIVE guidelines for animal care [35]. For human sera collection, the study followed the principles of the Helsinki Declaration (DoH 2024) of 1964, and informed consent, either written or by thumb printing, was obtained from each participant [36]. The study protocol was approved by the Ethics Committee of the Faculty of Medicine, Alexandria University (Permit Number: 0306484).

### Experimental animals

Swiss albino mice were used for maintaining parasitic life cycles. Mice were housed in appropriate cages under standard laboratory conditions (27 ± 2 °C; 70–80% humidity; 12 h light/dark cycle) with a standard pellet diet and water *ad libitum*.

### Laboratory maintenance of the parasitic life cycle

The life cycles of all parasites were maintained at the Department of Medical Parasitology, Faculty of Medicine, Alexandria University. The *S. mansoni* life cycle was maintained by serial passage between *Biomphalaria alexandrina* snails [37] and Swiss albino mice [38]. The *T. spiralis* life cycle was maintained through serial passage in Swiss albino mice by ingestion of live purified *T. spiralis* larvae [39]. The *T. gondii* life cycle was maintained by serial intraperitoneal (ip) passages of live tachyzoites of the virulent RH HXGPRT (-) strain in Swiss albino mice [40].

### Preparation of parasitic antigens

To prepare autoclaved antigens, cercariae of *S. mansoni*, larvae of *T. spiralis*, and tachyzoites of *T. gondii* were collected and autoclaved to generate A*Sm*A, A*Ts*A, and A*Tg*A, respectively. Cercariae of *S. mansoni* were collected by exposing infected snails for one hour to sunlight in dechlorinated water to allow the shedding of the cercariae [22]. Larvae of *T. spiralis* were collected by enzymatic digestion of skeletal muscles from *T. spiralis-*infected mice [23]. Tachyzoites of *T. gondii* were harvested from the peritoneal exudate of *T. gondii*-infected mice [24]. They were autoclaved separately in screw-capped glass vials at 121 °C, under pressure of 15 lb for 15 min. The antigens were kept at -20 °C for later usage [24]. Protein quantification was performed using a NanoDrop™ 2000 spectrophotometer (Thermo-scientific) and expressed in mg/ml.

### Anti-parasitic antibodies

#### Human sample collection

Non-cancerous patients visiting the Internal Medicine/Hepatology or Obstetrics and Gynecology outpatient clinics at Alexandria University Main and El-Shatby Hospitals, respectively, were the subjects of the study. Blood samples were collected from patients who were suspected of having schistosomiasis or toxoplasmosis, as well as from healthy controls. Sera were separated and stored at -20 °C for later usage.

#### Screening of serum samples for IgG-positive sera for *S. mansoni* and *T. gondii*

Collected serum samples were screened using ELISA kits for *S. mansoni-*specific IgG antibodies and *T. gondii-*specific IgG antibodies (MyBioSource Inc., San Diego, USA), Catalogue #: MBS495074 and MBS494548, respectively, according to the manufacturer’s instructions. Using an automated ELISA reader (Benchmark, BIO-RAD, California, U.S.A., Catalogue # 170–6850 Microplate Reader), the optical density of each sample was read at 450 nm within 15 min [41]. Samples with the highest titer were selected for the in vitro experimental study.

#### Preparation of IgG-positive serum for *T. spiralis*

Due to the scarce human infection with *T. spiralis* in Egypt, sera of mice chronically infected with *T. spiralis* (35 days post-infection) were used. An ELISA test was performed to confirm the positivity of *T. spiralis* IgG in the mice sera, as previously described [42]. In brief, wells of a 96-well plate were coated with diluted A*Ts*A, followed by an overnight incubation at 4 °C. 1% bovine albumin, used as a blocking buffer, was added to the plate, followed by incubation at room temperature for two hours. The wells were washed, and 100 µl of diluted mouse sera was added to the wells for two hours at 37 °C, followed by washing four times with phosphate buffer saline. A diluted secondary antibody (goat anti-mouse; Sigma-Aldrich) was added for one hour. Lastly, the plate was washed and incubated with the chromogenic substrate. The optical densities were read via an ELISA reader (BIO-RAD) at 450 nm within 15 min. Samples with the highest titer were selected for the in vitro experimental study [42].

### Cancer cells and culture

Frozen vials of different cancer cell lines (HT-29 human CRC cell line and HepG2 HCC cell line) were purchased from VACSERA Co., Cell Culture Unit, Dokky, Giza, Egypt, originally supplied from the American Type Culture Collection (ATCC). *Mycoplasma*-free cancer cells were cultured in high glucose Dulbecco’s Modified Eagle Medium (DMEM) (Sigma-Aldrich) supplemented with 10% fetal bovine serum (Sigma-Aldrich), 2 mM L-glutamine (Thermo Fisher Scientific), 1 mM sodium pyruvate (Fisher Scientific), Streptomycin (100 mg/ml) (Thermo Fisher Scientific) and Amphotericin-B (5 mg/ml) (Thermo Fisher Scientific) and maintained at the Tissue Culture Unit at Alexandria University.

### In vitro study protocol

#### Cancer cell maintenance

Cancer cell lines were cultured as previously described [43]. Five days before the experiment, cells were seeded in a sterile 24-well plate in complete culture media and incubated under a humidified atmosphere of 5% CO_2_ at 37 °C in an automated CO_2_ incubator. Cells were regularly checked using an inverted microscope until confluent.

#### Experimental protocol

One day before the experiment, cells were trypsinized, washed, resuspended in complete media, and plated in 96 microtiter plates [44]. Four sets of 96-well plates were prepared: two for each cell line. For the four plates, cells (HT-29 & HepG2) were cultured separately for 24 h and allowed to get confluent in a final volume of 100 µl of complete media/well (10,000 cells/well). The next day, the cells that did not attach and the excess liquid were discarded. A total of 100 µl of aliquoted media containing diluted antigens or serum was added [45].

In the first two plates, cells (HT-29 & HepG2) were treated with serial dilutions of parasitic antigens (A*Sm*A, A*Ts*A, and A*Tg*A) at 3.125, 6.25, 12.5, 25, 50, and 100 µg/ml concentrations [21, 46, 47]. In the other two plates, cells (HT-29 & HepG2) were exposed to serial dilutions of *S. mansoni*,* T. spiralis*, and *T. gondii* IgG-positive sera. Sera were used at a concentration of 2.5, 5, 10, 20, and 40% [45]. Negative control wells were included where cells were cultured either in media only or media treated with sera negative for parasite IgG antibodies. The experiment was performed in triplicate, and the data presented is the mean of the experimental replicates [21].

#### MTT protocol

For the assessment of cytotoxicity, the following protocol was applied. After 24 h incubation, 3-[4,5-dimethylthiazol-2-yl]-2,5-diphenyltetrazolium bromide (MTT) assay was used as previously described [21, 48]. Cells were incubated for the last two hours with 10 µl of MTT labelling agent (MTT formazan, 2 mg/ml; Sigma), added to each well according to the manufacturer’s instructions. One hundred µl of dimethyl sulfoxide (DMSO) (Sigma) was added to dissolve the formazan crystals, and the absorbance values were measured at 490 nm [49]. The proliferation was checked for each treatment, as well as the negative controls. To calculate the proliferation percentage, the absorbance of each treated group was divided by the absorbance of the media-only negative control wells and multiplied by 100. The following equation was applied: $\:(Atc/Anc)\times\:100$, where *Atc* is the absorbance of each treatment concentration and *Anc* is the absorbance of the media-only negative control. For the calculation of the percentage of inhibition in the antigen-treated groups, the proliferation percentage of each treatment concentration was subtracted from the proliferation percentage of the cells cultured in the media-only negative control group. To calculate the percentage of inhibition in the serum-treated groups, the proliferation percentage of each treatment concentration was subtracted from the proliferation percentage of the cells exposed to serum-negative for parasitic antibodies.

### Statistical analysis of the data

Data were analyzed using the IBM SPSS software package version 20.0. (Armonk, NY: IBM Corp). Quantitative data were expressed as mean and standard deviation. Student t-test was used to compare two groups for normally distributed quantitative variables, while the One-way ANOVA test was used for comparing the different studied groups, followed by the Post-Hoc test (Tukey) for pairwise comparison. Results were considered statistically significant at the 5% level.

## Results

### Effect of parasites’ immunomodulators on HT-29 colorectal cancer cell line

#### Effect of parasitic antigens on HT-29 colorectal cancer cell line

**Fig. 1:**
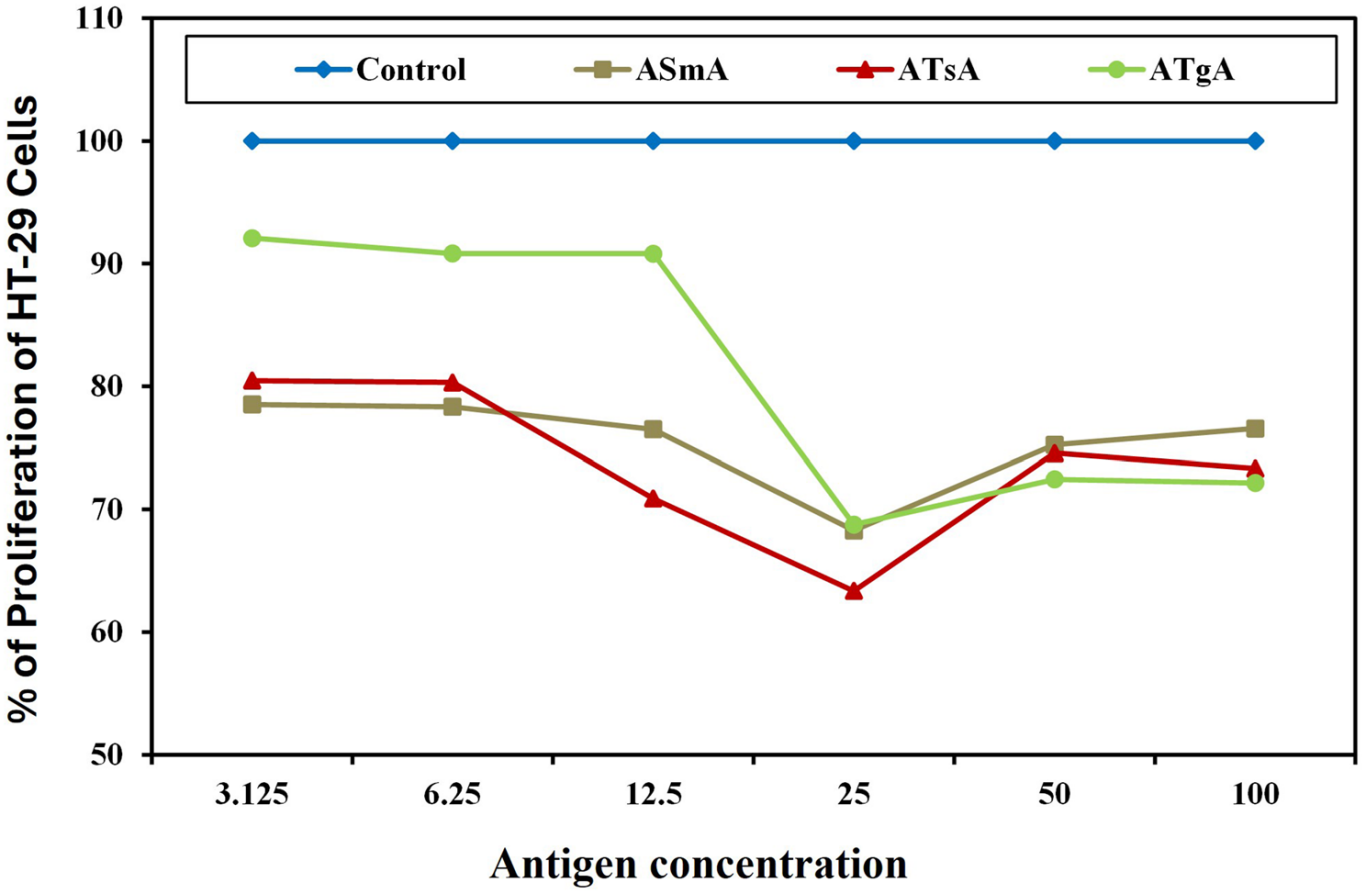
Graphical representation of HT-29 colorectal cancer cell proliferation percentage upon incubation with different concentrations of parasitic antigens for 24 h, compared to cells cultured in media only. Control: Cells cultured in media only, A*Sm*A: Cells incubated with autoclaved *Schistosoma mansoni* antigen, A*Ts*A: Cells incubated with autoclaved *Trichinella spiralis* antigen, A*Tg*A: Cells incubated with autoclaved *Toxoplasma gondii* antigen. HT-29 colorectal carcinoma cells were incubated with serial concentrations of different parasitic antigens for 24 h, and the proliferation percentage was measured using the MTT assay

Exposure of HT-29 colorectal cancer cells to serial concentrations of parasitic antigens (A*Sm*A, A*Ts*A, and A*Tg*A) for 24 h induced a statistically significant reduction (P˂ 0.05) in colorectal cancer cell proliferation at all concentrations tested compared to untreated colorectal cancer cells. The maximum inhibitory percentage was achieved at 25 µg/ml for all parasitic antigens, A*Sm*A, A*Ts*A, and A*Tg*A, with percentages of inhibition of 31.72%, 36.65%, and 31.23% respectively. The highest significant inhibition percentage was demonstrated for A*Ts*A (36.65%), which was significantly higher than A*Sm*A and A*Tg*A. Additionally, no significant difference was observed between A*Sm*A and A*Tg*A. While the three antigens induced significant inhibitory effects at all concentrations, a noteworthy finding was that at higher concentrations (50 µg/ml and 100 µg/ml), a significant proliferative effect was observed compared to the 25 µg/ml concentration for all antigens, with no significant difference between these two higher concentrations for all tested antigens (Fig. 1, Suppl. Table 1).

#### Effect of serum positive for parasitic IgG on HT-29 colorectal cancer cell line

**Fig. 2:**
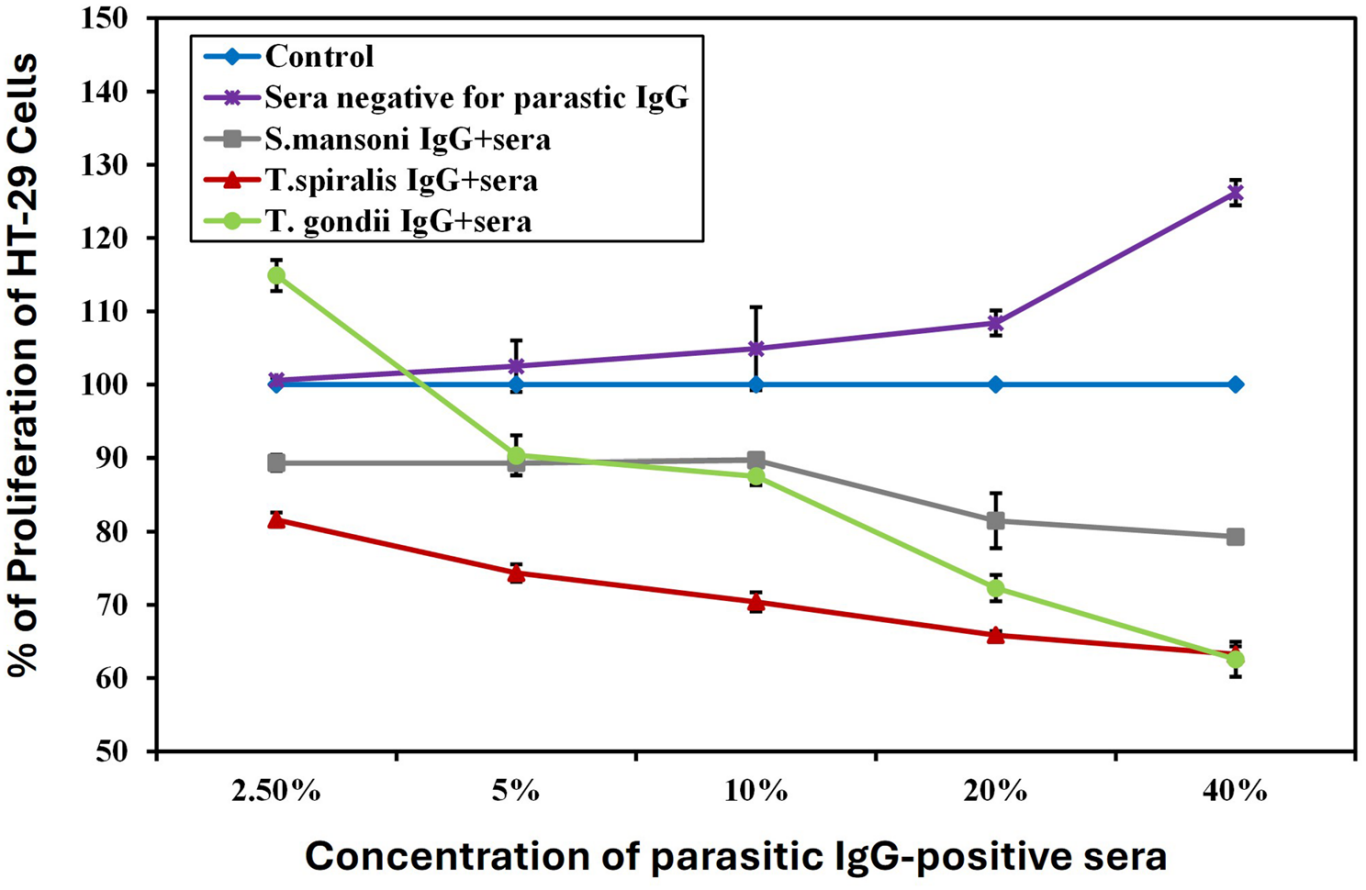
Graphical representation of HT-29 colorectal cancer cell proliferation percentage upon incubation with different concentrations of sera positive/ negative for parasitic IgG for 24 h, compared to cells cultured in media only. Control: Cells cultured in media only, Sera negative for parasitic IgG: Cells incubated with sera negative for parasitic IgG, *S. mansoni* IgG^+^ sera: Cells incubated with sera positive for *Schistosoma mansoni* IgG, *T. spiralis* IgG ^+^ sera: Cells incubated with sera positive for *Trichinella spiralis* IgG, *T. gondii* IgG ^+^ sera: Cells incubated with sera positive for *Toxoplasma gondii* IgG. HT-29 colorectal cancer cells were incubated with serial concentrations of different sera positive for parasitic antibodies for 24 h, and the proliferation percentage was measured using the MTT assay

Exposure of HT-29 colorectal cancer cells to serial concentrations of serum positive for parasitic antibodies for 24 h induced a statistically significant inhibition in cell proliferation in a concentration-dependent manner compared to both the untreated and the negative sera controls (P˂ 0.05). The inhibitory effect was significant starting from 2.5% for *S. mansoni* and *T. spiralis* IgG-positive sera, and 5% for *T. gondii* IgG-positive sera, up to 40% for all parasitic antibodies. The maximum inhibitory percentage was observed at 40% for both *T. spiralis* and *T.gondii* IgG-positive sera, which showed comparable efficacy with percentages of inhibition of 49.90% and 50.43%, respectively, significantly higher than that exposed to *S. mansoni* IgG-positive sera (37.17%) (Fig. 2, Suppl Table 2).

Notably, *T. gondii* IgG-positive sera at a low concentration of 2.5% had a biphasic effect on HT-29 colorectal cancer cells, promoting cell proliferation compared to cells exposed to media only and cells exposed to negative sera controls. Additionally, negative sera for parasite IgG antibodies promoted significant proliferation of HT-29 colorectal cancer cells compared to the untreated control at a concentration ranging from 20% to 40% (Fig. 2, Suppl Table 2).

### Effect of parasites’ immunomodulators on HepG2 hepatocellular carcinoma cell line

#### Effect of parasitic antigens on HepG2 hepatocellular carcinoma cell line

**Fig. 3:**
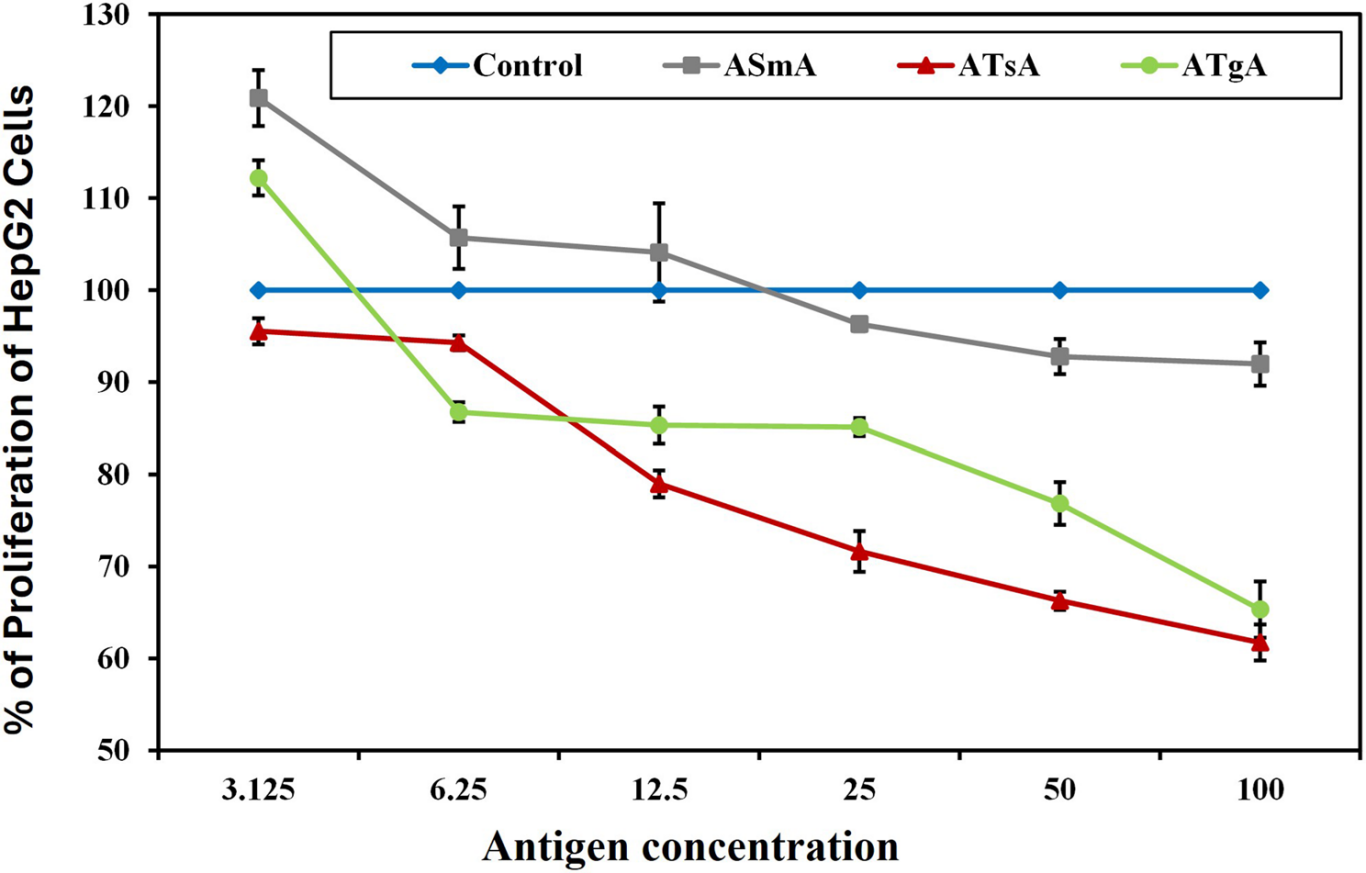
Graphical representation of HepG2 hepatocellular carcinoma cell proliferation percentage upon incubation with different concentrations of parasitic antigens for 24 h, compared to cells cultured in media only. Control: Cells cultured in media only, A*Sm*A: Cells incubated with autoclaved *Schistosoma mansoni* antigen, A*Ts*A: Cells incubated with autoclaved *Trichinella spiralis* antigen, A*Tg*A: Cells incubated with autoclaved *Toxoplasma gondii* antigen. HepG2 hepatocellular carcinoma cells were incubated with serial concentrations of different parasitic antigens for 24 h, and the proliferation percentage was measured using the MTT assay

Exposure of HepG2 hepatocellular carcinoma cells to serial concentrations of parasitic antigens (A*Sm*A, A*Ts*A, and A*Tg*A) for 24 h resulted in a concentration-dependent decrease in cell proliferation. The inhibitory effect was observed at concentrations starting from 25 µg/ml for A*sm*A, 6.25 µg/ml for A*Ts*A, and 6.25 µg/ml for A*Tg*A up to 100 µg/ml for all antigens. The maximum inhibitory percentage was achieved at 100 µg/ml for all antigens, with A*Ts*A showing a 38.27%, A*Tg*A with a 34.68%, and A*Sm*A with an 8.03% inhibition percentage. A*Ts*A and A*Tg*A had similar inhibitory effects, both significantly higher than A*Sm*A. Remarkably, A*Sm*A and A*Tg*A at a low concentration of 3.125 µg/ml were noticed to have a biphasic effect on HepG2 hepatocellular carcinoma cells, as they induced significant cell proliferation (Fig. 3, Suppl. Table 3).

#### Effect of serum positive for parasitic IgG on HepG2 hepatocellular carcinoma cell line

Exposure of HepG2 hepatocellular carcinoma cells to serial concentrations of sera positive for *S. mansoni*, *T. spiralis*,* and T. gondii* IgG for 24 h significantly inhibited cell proliferation in a concentration-dependent manner. The inhibition started at 2.5% for *T. gondii* IgG-positive sera, 5% for *T. spiralis* IgG-positive sera, and 10% for *S. mansoni* IgG-positive sera, up to 40% compared to negative sera control.

**Fig. 4:**
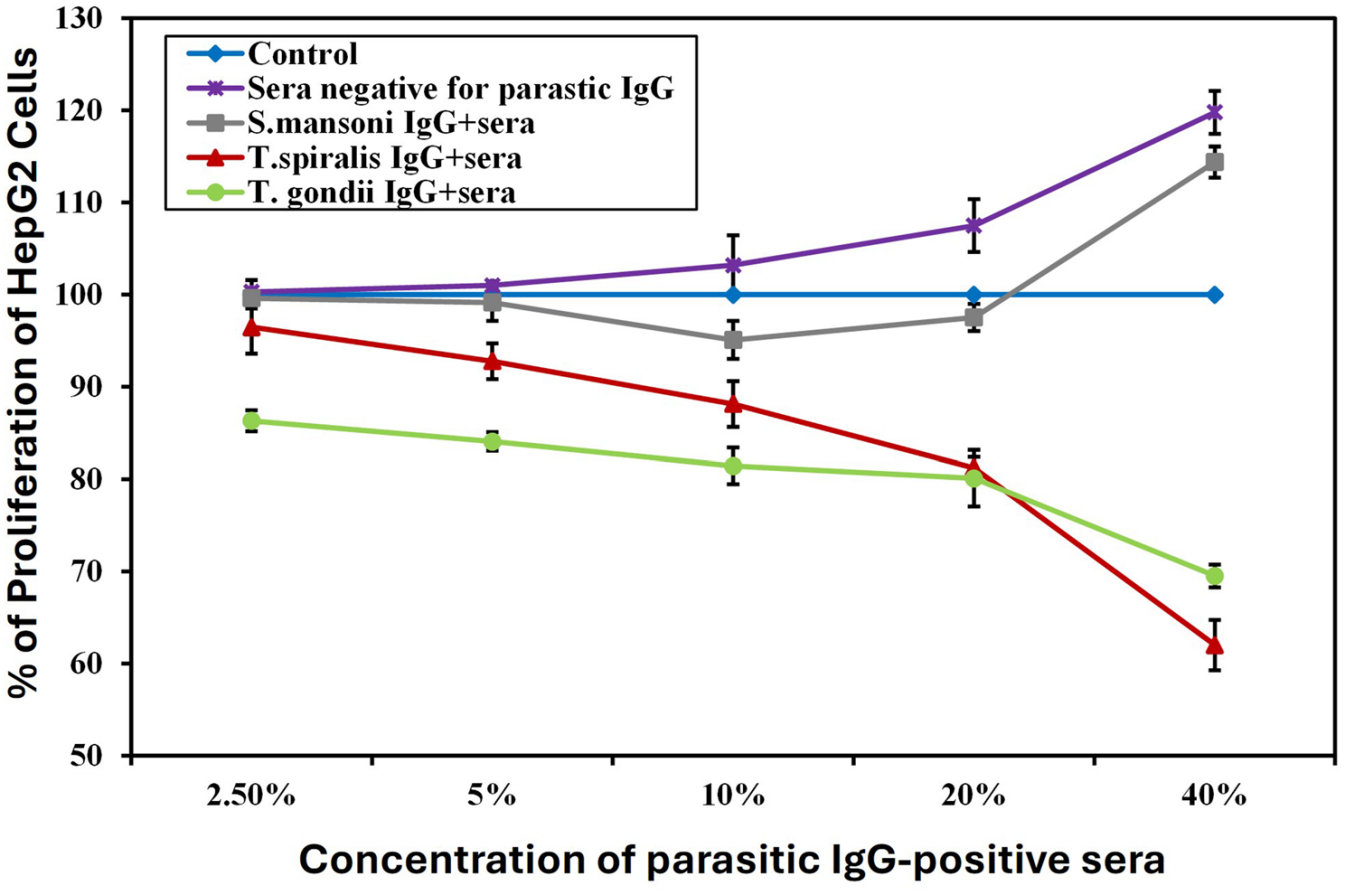
Graphical representation of hepatocellular carcinoma cell proliferation percentage upon incubation with different concentrations of sera positive/ negative for parasitic IgG for 24 h, compared to cells cultured in media only. Control: Cells cultured in media only, Sera negative for parasitic IgG: Cells incubated with sera negative for parasitic IgG, *S. mansoni* IgG^+^ sera: Cells incubated with sera positive for *Schistosoma mansoni* IgG, *T. spiralis* IgG ^+^ sera: Cells incubated with sera positive for *Trichinella spiralis* IgG, *T. gondii* IgG ^+^ sera: Cells incubated with sera positive for *Toxoplasma gondii* IgG. HepG2 hepatocellular carcinoma cells were incubated with serial concentrations of sera positive for different parasitic antibodies for 24 h, and the proliferation percentage was measured using the MTT assay

**Fig. 5:**
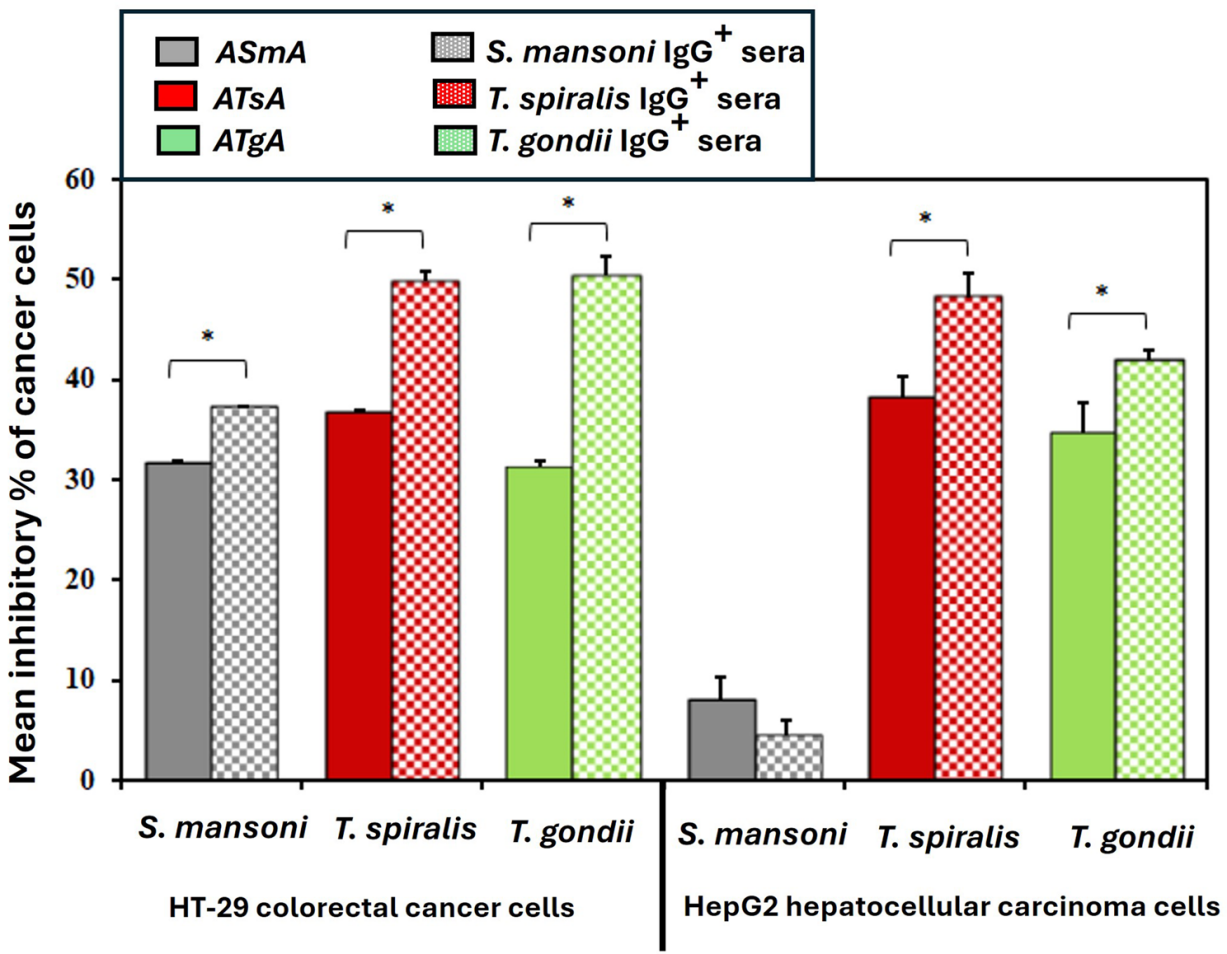
Graphical representation of the maximal inhibitory percentage of HT-29 colorectal and HepG2 hepatocellular carcinoma cells upon incubation with parasitic antigens and their corresponding parasitic IgG-positive sera for 24 h. A*Sm*A: Cells incubated with autoclaved *Schistosoma mansoni* antigen, A*Ts*A: Cells incubated with autoclaved *Trichinella spiralis* antigen, A*Tg*A: Cells incubated with autoclaved *Toxoplasma gondii* antigen. *S. mansoni* IgG^+^ sera: Cells incubated with sera positive for *Schistosoma mansoni* IgG, *T. spiralis* IgG ^+^ sera: Cells incubated with sera positive for *Trichinella spiralis* IgG, *T. gondii* IgG ^+^ sera: Cells incubated with sera positive for *Toxoplasma gondii* IgG. HT-29 colorectal cancer cells and HepG2 hepatocellular carcinoma cells were incubated with serial concentrations of different parasitic antigens/ parasitic IgG-positive sera for 24 h, and the inhibitory percentages were measured using the MTT assay. *: Represents significance between the inhibitory percentages of the examined concentrations of parasitic antigens and their corresponding IgG^+^ sera

The maximum inhibitory percentage was observed at a concentration of 40% for *T. spiralis* IgG-positive sera (48.25%), which was statistically significantly higher than cells exposed to both *T.gondii* IgG-positive (41.98%) and *S.mansoni* IgG-positive sera (4.54%). Although *S. mansoni* IgG-positive sera at a concentration of 40% showed inhibition in cancer cell proliferation as compared to negative sera control, this value was significantly higher than that of the untreated cells cultured in media alone. Remarkably, negative sera for parasite IgG antibodies induced significant cell proliferation compared to the untreated control, starting from 20% to 40%. (Fig. 4, Suppl. Table 4). Notably, among all tested parasites’ immunomodulators, anti-*T. spiralis* and *T. gondii* antibodies exhibited the maximum inhibitory effect on both cancer cell lines (Fig. 5, Suppl. Table 5).

## Discussion

Parasites and their derivatives were proven to alleviate cancer in experimental models via multiple strategies [7, 29, 32, 33]. While the immunomodulatory mechanism constitutes a main strategy in fighting cancer, other verified mechanisms are also recognized, including their direct cytotoxic capacity, inhibition of neoangiogenesis, supplemented by the molecular mimicry theory [12, 15, 29, 33, 50–52]. However, rather than utilizing a systematic platform for the selection process, previous studies have utilized a random selection pattern to match parasites with different cancer types, except for some studies that screened for the molecular homology between cancer cells and parasites [28, 42, 53, 54]. Therefore, there are gaps in configuring which parasites may be linked to specific cancer types. Our objective was to identify potentially potent PIMs that may have antineoplastic potency. This mix-and-match selective strategy can assist researchers in designing a long-term road map for the development of potent novel cancer therapeutics of parasitic origin. Several parasitic antigens were documented to have antineoplastic activity both in vitro and in vivo [7, 12]. However, the role of parasitic antibodies in combating cancer remains underexplored. Enforced by the molecular mimicry theory between parasites and cancer cells, antibodies that develop against parasites can potentially attack cancer cells. This may explain the restricted cancer development in seropositive patients for parasitic antibodies [55].

In this context, the current study investigated the antiproliferative effect of six different parasites’ immunomodulators (antigens and antibodies) of *S. mansoni*, *T. spiralis*, and *T. gondii* on human cancer cell lines (HT-29 CRC and HepG2 HCC cells). These parasites were selectively chosen due to their previous records of antineoplastic activity experimentally [7]. Correspondingly, these cancer types were specifically selected due to their high incidence and severe health consequences [2, 4].

### In vitro antineoplastic potential of parasites’ immunomodulators on human HT-29 colorectal cancer cell line

#### *Schistosoma mansoni* immunomodulators

There is conflicting evidence regarding the link between *S. mansoni* and colon cancer in both human and experimental studies [56]. Interestingly, emerging reports have shown the antineoplastic properties of *S. mansoni* and *S. japonicum* in different experimental cancer models [32, 33, 52, 57, 58].

The results of our study showed that in vitro exposure of HT-29 colorectal cells to A*Sm*A induced a significant inhibition of colorectal cell proliferation at all tested concentrations compared to the untreated control, with the maximum inhibitory percentage of 31.72% achieved at a concentration of 25 µg/ml. Our results are in agreement with other in vitro results, which reported in vitro antineoplastic activity for *S. mansoni* egg and cercarial antigens on colorectal cancer cell lines (HCT-116 and DLD-1) and MCF-7 human breast cancer cell line [32, 59]. Additionally, in vivo studies reported the antineoplastic activity of *S. mansoni* in a murine cancer model [60], transplanted sarcoma model [61], colon, colorectal, and breast cancer murine models [32, 33, 59]. The antineoplastic activity of *Schistosoma* is believed to involve an immunomodulatory mechanism, potentially through molecular mimicry between *S. mansoni* and cancer cells [33]. This mimicry has been observed between *S. mansoni* and MCF-7 human breast cancer and A549 lung cancer cells at an approximate molecular weight of 80 kDa [42].

Additionally, our study demonstrated that *S. mansoni* IgG-positive human sera inhibited colorectal cancer cell proliferation in a concentration-dependent manner, with the maximum significant inhibitory percentage of 37.17%, noticed at a concentration of 40%. To the best of our knowledge, this is the first study highlighting the antineoplastic activity of anti-S. *mansoni* antibodies against colorectal cancer. Notably, negative sera for parasitic IgG antibodies induced significant proliferation of HT-29 colorectal cancer cells compared to untreated cells. This aligns with previous research showing that human serum promotes cell growth and migration [62, 63].

#### *Trichinella spiralis* immunomodulators

Our study revealed an in vitro antineoplastic activity of *T. spiralis* immunomodulators against HT-29 colorectal cancer cells. Exposure of colorectal cancer cells to A*Ts*A and *T. spiralis-*IgG-positive mice sera induced a significant reduction in cell proliferation at all concentrations tested, with the maximum inhibitory percentage of 36.65% at 25 µg/ml and 49.90% at 40% for both A*Ts*A and *T. spiralis* IgG-positive sera, respectively. Our results align with a previous in vivo study showing a diminishing in colorectal carcinoma growth in mice infected with *T. spiralis* [64]. However, another in vivo study using a DMH-induced colon carcinogenesis murine model showed conflicting results [33]. Interestingly, that study reported a notable increase in the average lesion size compared to the control group. The authors suggested that the initial immune-stimulant response of *T. spiralis* antigen, including mucosal immune responses, may have interacted with the inflammatory effects induced by DMH, leading to the observed outcome [33]. Therefore, further research is necessary to investigate the in vivo anti-cancerous properties of *T. spiralis* antigens in colorectal cancer xenograft animal models.

Additionally, our finding of the significant inhibitory effect of *T. spiralis* IgG-positive sera on colorectal cancer cell proliferation further documents its antineoplastic activity. Previous studies have shown similar effects of anti-*T. spiralis* antiserum and antiserum prepared against TPD52, a shared antigen between *T. spiralis* and osteosarcoma, on osteosarcoma cells [54]. The inhibitory effect reported with *T. spiralis* IgG-positive on colorectal cells may postulate the existence of shared antigens between *T. spiralis* and colorectal cancer cells, thus warranting further investigation. Notably, shared antigens were demonstrated between *T. spiralis* and human cancer cell lines (MCF-7 breast and A549 lung cancer) at approximate molecular weights of 70 and 35 kDa [42], which could partially justify *T. spiralis’* potent antineoplastic effects in breast cancer [65, 66] and lung cancer murine models [67, 68]. Our study is considered the first report of the antineoplastic activity of *T. spiralis* immunomodulators against colorectal cancer cells.

#### *Toxoplasma gondii* immunomodulators

In our in vitro study, A*Tg*A and *T. gondii* IgG-positive human sera significantly reduced the colorectal cancer cells’ proliferation at all concentrations tested, with the maximum inhibitory percentage observed at a concentration of 25 µg/ml and 40%, respectively. This is the first study demonstrating the antineoplastic activity of anti-*T. gondii* antibodies against colorectal cancer. These findings are consistent with previous studies that demonstrated apoptosis of HCT116 human CRC cells incubated in vitro with *T. gondii* recombinant proteins, such as GRA16 [69], and GRA8 [70].

Remarkably, *T. gondii* has shown promising anti-cancerous effects against nearly all types of cancer studied so far [7]. For instance, attenuated *T. gondii* strain (RH-ΔGRA17) significantly slowed tumor growth in a mouse model of colon adenocarcinoma (MC38) [71]. *Toxoplasma gondii* lysate antigens [72], derivatives such as *T. gondii* GRA8 [70], *T. gondii* profilin-like protein [73], and *T. gondii* rGRA6Nt protein [74], as well as exosomes derived from dendritic cells infected with *T. gondii* [75, 76] significantly inhibited tumor growth in several murine models of CRC.

It is worth noting that in our study, the three examined antigens at higher concentrations of 50 µg/ml and 100 µg/ml induced a significant proliferative effect on HT-29 colorectal cancer cells compared to the 25 µg/ml concentration, with no significant difference between them. Despite this, the antineoplastic activity of the three parasitic antigens at these concentrations was still significantly lower compared to untreated cells. This relative increase within the inhibited range may be due to the heterogeneous nature of the HT-29 colorectal cell line, resulting in more proliferating cells compared to lower concentrations [77], although the proliferations are still inhibited compared to the untreated cells.

Collectively, our study is in agreement with other studies that demonstrated the in vitro antineoplastic activity of other parasites and their derivatives on colon cancer cells, both in vitro and in vivo, such as *E. granulosus* [78–80], *Toxocara canis (T. canis)* [47], *Heligmosomoides polygyrus* [81], *Plasmodium* [82, 83] among others [84–87].

### In vitro antineoplastic potential of parasites’ immunomodulators on human HepG2 hepatocellular carcinoma cell line

#### *Schistosoma mansoni* immunomodulators

A*Sm*A and *S. mansoni* IgG-positive human serum induced a significant inhibitory effect on HepG2 human HCC cell proliferation, with the highest inhibitory percentage at 100 µg/ml and 40% respectively. Our results align with previous studies demonstrating the in vitro antineoplastic potential of various parasites and their derivatives against different HCC cell lines, such as *S. japonicum* [52, 88–90].

Contrary to our results, other studies linked liver cancer and *S. mansoni* infection, possibly exacerbated by hepatitis viruses B and C, which cumulatively may induce suppression in cell-mediated immunity and promote liver cancer [91, 92]. Studies in liver cancer animal models have shown that *S. mansoni* infection can lead to liver dysplastic changes and an aggressive cancer pattern [93]. Furthermore, antigens secreted by *S. mansoni* have been found to activate human and hamster liver cells by inducing c-Jun and STAT3, crucial regulators of liver cancer [94].

#### *Trichinella spiralis* immunomodulators

In our study, a significant reduction in liver cancer cell (HepG2) proliferation was observed upon exposure to A*Ts*A and *T. spiralis* IgG-positive mice sera in a concentration-dependent manner, with the highest significant inhibition observed at a concentration of 100 µg/ml and 40%, respectively. This is the first report supporting the antineoplastic activity of anti-*T. spiralis* antibodies against HCC. This is consistent with previous in vitro studies that reported the inhibitory effect of *T. spiralis* antigens, derivatives, and proteins encoded by *T. spiralis* on various cancer cell lines, including ascitic hepatoma (H22), hepatoma (H7402) [46, 95] and HepG2 cells [96].

Similarly, in vivo experimental studies have also shown that infecting mice with *T. spiralis* could inhibit the growth of ascitic hepatoma H22 [46] and Hepatoma Hep1-6 carcinoma growth [97], as well as slow tumor progression by upregulating the expression of the proapoptotic marker Bcl-2 and improving survival in mice [98]. *Trichinella spiralis* has also been shown to induce apoptosis in HCC by utilizing regulatory pathways similar to those involved in cancer cell apoptosis, offering a potential strategy for combating this malignancy [99].

Our results regarding the antineoplastic activity of *T. spiralis* immunomodulators against human cancer cell lines (HT-29 CRC and HepG2 HCC) are consistent with other in vitro studies on small and non-small lung cancer cells (H446, A549) [100–102], human acute and chronic myeloid leukemia (K562, U937, R1) [95, 103, 104], EL-4 lymphoma [105], allogenic P815 mastocytoma [105], S180 sarcoma [95], B16 melanoma [106], and human cervical carcinoma HeLa cells [107]. Our results are also consistent with the in vivo antineoplastic activity of *T. spiralis* infection and its derivatives in murine forestomach carcinoma [95], lung cancer [67, 68], breast cancer [65, 66], multiple myeloma [108], sarcoma [95, 109, 110], osteosarcoma [54], melanoma [106, 111–113], and glioma cancer models [114]. To the best of our knowledge, our study is the first to report the in vitro antineoplastic activity of anti-*T. spiralis* antibodies against HCC.

#### *Toxoplasma gondii* immunomodulators

Similarly, A*Tg*A and *T. gondii IgG-positive* human sera induced a significant inhibition of HepG2 HCC cell proliferation in a concentration-dependent manner, with the maximum inhibitory percentage achieved at 100 µg/ml and 40% respectively. This synchronizes with previous studies utilizing *T. gondii* tachyzoites (RH strain) and derivatives on H7402 HCC cells [115], *T. gondii* GRA16 on HepG2 HCC cells [116] and *T. gondii* GRA15II-polarized macrophages on Hepa1-6 cells [117]. These findings were further supported by in vivo studies where *T. gondii* GRA16 and *T. gondii* GRA15II-polarized macrophages significantly reduced tumor size in HCC murine models [116, 117].

Furthermore, our study demonstrated that *T. gondii* IgG-positive human sera induced a significant inhibition of HepG2 HCC cell proliferation across all tested concentrations in a concentration-dependent manner. To the best of our knowledge, this is the first report of the antineoplastic activity of anti-*T. gondii* antibodies against HCC. Our result aligns with and supports the study by Seyedeh et al., 2015, which concluded that individuals with low anti-*Toxoplasma* antibody levels exhibited resistance to cancer [55].

On the other hand, contradictory results have been reported regarding *Toxoplasma* seropositivity in cancer patients. It has been suggested that approximately 40% of cancer patients have *Toxoplasma* IgG antibodies in their sera [118–121]. Some studies reported significantly elevated *Toxoplasma* IgG levels in cancer patients, with higher titers associated with a more advanced cancer grade [122–124]. Moreover, toxoplasmosis has been linked to higher mortality rates in different types of cancers, including Hodgkin’s lymphoma, leukemia, melanoma, and brain cancers [118, 121, 125]. However, these associations may be attributed to the immunosuppressive effects of cancer and the intake of chemotherapy, which increase susceptibility to opportunistic infections such as toxoplasmosis [126].

Our results for the antineoplastic activity of *T. gondii* immunomodulators against human cancer cell lines (HT-29 CRC and HepG2 HCC) are in line with other in vitro studies on BGC-823 gastric and esophageal squamous cell carcinoma (EC109) [127, 128], mammary cancer cell line (TUBO cells) [129], MCF-7 human breast cancer cells [48], and non-small lung cancer cells (A549) [128]. This is in addition to murine lymphoma and mastocytoma cell lines [130], fibrosarcoma WEHI-164 cells [131], ovarian cell lines (A2780 & A2780-CP) [132], DU-145 prostate cell line [128], and human glioma U373MG & U87MG cells [21]. In vivo studies have further confirmed the antineoplastic activity of *T. gondii* in pancreatic cancer [14, 17, 133–135], mammary tumors [136–138], lung cancer [71, 139, 140], sarcoma [136, 141–144], melanoma [15, 18, 71, 145–147], ovarian tumors [15, 148], glioma [21], ependymoblastoma [149], and thymoma murine models [150].

Notably, the current study revealed a significant in vitro antineoplastic activity of *S. mansoni*, *T. spiralis*, and *T. gondii* IgG-positive sera against HT-29 CRC cells and HepG2 HCC cells. Our results align with previous in vitro experimental studies in the current era, where studies demonstrated that anti-hydatid cyst antibodies exhibited antineoplastic activity on NCI-H209/An1 human small-cell lung cancer cells [45] and 4T1 breast cancer cell line [151]. Parasitic antibodies have been found to selectively attach to various cancer cell lines, supporting the theory of molecular mimicry. For example, anti-*T.cruzi* cross-reacted with human mammary cancer cells (T47D and MCF-7 cell lines), human colon cells [85] and acute lymphoblastic leukemia cells [152]. Additionally, *T. cruzi* proteins, as well as antibodies against both *T. canis* and *Acanthamoeba*, cross-reacted with neuroblastoma cells [152, 153]. Interestingly, both anti-*T. cruzi* and anti-hydatid cyst antibodies reacted with lung cancer cells [154, 155]. Surprisingly, only the anti-*T. gondii* antiserum showed significant reactivity with mouse melanoma cells, while antibodies from other parasites did not [156]. However, it should be reported that various parasites have demonstrated in vivo antineoplastic activity in hepatoma and HCC, such as *S. japonicum* [52, 88–90], *Plasmodium berghei* [157], *Plasmodium yoelii* [19, 158], *Angiostrongylus cantonensis* [159] and *Setaria equine* [51, 160].

Notably, in the current study, at a low concentration of 3.125 µg/ ml of A*Sm*A and A*Tg*A and a low concentration of 2.5% for *T. gondii* IgG-positive sera, a biphasic effect was demonstrated on HepG2 HCC cells and HT-29 CRC cells, respectively, where significant cellular proliferation was observed. This biphasic response may suggest interaction with multiple cellular targets or signalling pathways. It could also be attributed to hormesis phenomena, receptor dynamics, or disruption of cellular homeostasis [161]. Therefore, our study emphasizes the importance of mechanistic investigations of parasites’ immunomodulators’ antineoplastic activity in pharmacological and toxicological studies.

Additionally, there is an essential need for in-depth investigational studies to establish a concrete foundation for selective identification of parasite/cancer types. Further elaborative studies should focus on purification of parasitic antibodies for further identification, as well as molecular characterization and identification of the most immunogenic antigenic components of the autoclaved parasitic antigens for later clinical applicability. This could lead to the development of nature-based anti-cancer candidates of parasitic origin for targeting cancer.

## Conclusion

The perception of the value of parasites has been entirely switched due to their potential anti-cancerous effects observed in experimental studies. Collectively, this study demonstrated that *S. mansoni*, *T. spiralis*, and *T. gondii* immunomodulators have significant antineoplastic effects on HT-29 colorectal and HepG2 hepatocellular carcinoma cells. The highest antineoplastic activity was reported upon using *T. spiralis* immunomodulators and anti-*T. gondii* antibodies for colorectal cancer cells. For hepatocellular carcinoma cells, *T. spiralis* immunomodulators and *T.gondii* antigen demonstrated the highest antineoplastic activity. These findings could enlighten the path for promising cancer-fighting strategies. This could lead to the development of nature-based candidates of parasitic origin targeting cancer for future research and clinical applications.

## Supplementary Information

Below is the link to the electronic supplementary material.

Supplementary Material 1

## Data Availability

No datasets were generated or analysed during the current study.

## References

[1] Siegel RL, et al. Cancer statistics, 2025. CA Cancer J Clin. 2025;75(1):10–45. 39817679 10.3322/caac.21871 PMC11745215

[2] Danpanichkul P, et al. Epidemiology of Gastrointestinal cancers: a systematic analysis from the global burden of disease study 2021. Gut. 2025;74(1):26–34. 10.1136/gutjnl-2024-333227 39242191

[3] Arnold M, et al. Global burden of 5 major types of Gastrointestinal cancer. Gastroenterology. 2020;159(1):335–e34915. 32247694 10.1053/j.gastro.2020.02.068 PMC8630546

[4] Kinsey E, Lee HM. Management of hepatocellular carcinoma in 2024: the multidisciplinary paradigm in an evolving treatment landscape. Cancers (Basel). 2024;16(3). 10.3390/cancers16030666 PMC10854554 38339417

[5] Waldman AD, Fritz JM, Lenardo MJ. A guide to cancer immunotherapy: from T cell basic science to clinical practice. Nat Rev Immunol. 2020: pp. 1–18. 10.1038/s41577-020-0306-5 PMC7238960 32433532

[6] Fasihi-Karami M, et al. Association between some helminths and tumorigenesis through immunological and biochemical factors. Curr Cancer Ther Rev. 2023;19(2):96–102.

[7] Eissa MM, Salem AE, El N, Skhawy. Parasites revive hope for cancer therapy. Eur J Med Res. 2024;29(1):1–56. 39367471 10.1186/s40001-024-02057-2 PMC11453045

[8] Eissa MM, El-Faham MH, Skhawy NE. Bridging the gap for diverse applications of parasites as advanced cancer therapeutics: current progress and future directions. Infect Agent Cancer. 2025;20(1):53. 40731364 10.1186/s13027-025-00679-7 PMC12309174

[9] Yazdanbakhsh M, Matricardi PM. Parasites and the hygiene hypothesis: regulating the immune system? Clin Rev Allergy Immunol. 2004;26:15–23. 14755072 10.1385/CRIAI:26:1:15

[10] Akgul H, et al. Echinococcus against cancer: why not? Cancer. 2003;98(9):1995–9. 14584087 10.1002/cncr.11752

[11] Garcia SB, et al. A retrospective study of histopathological findings in 894 cases of megacolon: what is the relationship between megacolon and colonic cancer? Rev Inst Med Trop Sao Paulo. 2003;45:91–3. 12754574 10.1590/s0036-46652003000200007

[12] Darani HY, Yousefi M. Parasites and cancers: parasite antigens as possible targets for cancer immunotherapy. Future Oncol. 2012;8(12):1529–35. 23231515 10.2217/fon.12.155

[13] Badri-Chookami M, et al. Effect of alive Protoscoleces of hydatid cyst on the growth of melanoma cells in mouse model. I U M S. 2014;32(281):486–92.

[14] Bahwal SA, et al. Attenuated Toxoplasma gondii enhances the antitumor efficacy of anti-PD1 antibody by altering the tumor microenvironment in a pancreatic cancer mouse model. J Cancer Res Clin Oncol. 2022;148(10):2743–57. 35556163 10.1007/s00432-022-04036-8 PMC11800998

[15] Baird JR, et al. Avirulent Toxoplasma gondii generates therapeutic antitumor immunity by reversing immunosuppression in the ovarian cancer microenvironment. Cancer Res. 2013;73(13):3842–51. 23704211 10.1158/0008-5472.CAN-12-1974 PMC3702636

[16] Chen L, et al. Antitumor effect of malaria parasite infection in a murine Lewis lung cancer model through induction of innate and adaptive immunity. PLoS ONE. 2011;6(9):e24407. 21931708 10.1371/journal.pone.0024407 PMC3170332

[17] Fox BA, et al. Targeting tumors with nonreplicating Toxoplasma gondii uracil auxotroph vaccines. Trends Parasitol. 2013;29(9):431–7. 23928100 10.1016/j.pt.2013.07.001 PMC3777737

[18] Li Y, et al. Antitumor effects of a Toxoplasma mutant lacking lactate dehydrogenases. Parasitol Res. 2021;120:3335–9. 34405281 10.1007/s00436-021-07283-9

[19] Liang Y, et al. Plasmodium infection prevents recurrence and metastasis of hepatocellular carcinoma possibly via Inhibition of the epithelial–mesenchymal transition. Mol Med Rep. 2021;23(6):1–10. 10.3892/mmr.2021.12057 PMC8025467 33846776

[20] Ranasinghe SL, et al. Kunitz type protease inhibitor EgKI-1 from the canine tapeworm Echinococcus granulosus as a promising therapeutic against breast cancer. PLoS ONE. 2018;13(8):e0200433. 30169534 10.1371/journal.pone.0200433 PMC6118354

[21] Choo J-D. Inhibitory effects of Toxoplasma antigen on proliferation and invasion of human glioma cells. J Korean Neurosurg Soc. 2005;37:129–36.

[22] Eissa MM, et al. Autoclaved cercarial vaccine: a new hope against schistosomiasis parasitologic, histopathologic and Immunologic studies. J Egypt Soc Parasitol. 1998;28(2):461–79. 9707675

[23] Eissa MM, et al. Vaccination trial against experimental trichinellosis using autoclaved Trichinella spiralis larvae vaccine (ATSLV). J Egypt Soc Parasitol. 2003a;33(1):219–28. 12739813

[24] Eissa MM, et al. Initial characterization of an autoclaved Toxoplasma vaccine in mice. Exp Parasitol. 2012;131(3):310–6. 22595548 10.1016/j.exppara.2012.05.001

[25] Khalil EAG, et al. Safety and immunogenicity of a candidate vaccine for visceral leishmaniasis (Alum-precipitated autoclaved leishmania major + BCG) in children: an extended phase II study. Ann Trop Paediatr. 2006;26(4):357–61. 17132302 10.1179/146532806X152890

[26] Tosyali OA, et al. Nano-co-delivery of lipophosphoglycan with soluble and autoclaved leishmania antigens into PLGA nanoparticles: evaluation of in vitro and in vivo immunostimulatory effects against visceral leishmaniasis. Mater Sci Eng C. 2021;120:111684. 10.1016/j.msec.2020.111684 33545846

[27] Eissa MM, et al. Further studies on autoclaved cercarial vaccine against schistosomiasis: safety, longevity and stability. J Egypt Soc Parasitol. 2003;33(2):541–60. 14964666

[28] Eissa MM, et al. Prophylactic antineoplastic activity of Toxoplasma gondii RH derived antigen against Ehrlich solid carcinoma with evidence of shared antigens by comparative Immunoblotting. Infect Agents Cancer. 2023;18(21):1–13. 10.1186/s13027-023-00500-3 PMC10082516 37029378

[29] Ismail CA, et al. Toxoplasma gondii–derived antigen modifies tumor microenvironment of Ehrlich solid carcinoma murine model and enhances immunotherapeutic activity of cyclophosphamide. Med Oncol. 2023;40:1–13. 10.1007/s12032-023-01994-y PMC10073061 37014499

[30] Eissa MM, et al. Anti-arthritic activity of schistosoma mansoni and Trichinella spiralis derived-antigens in adjuvant arthritis in rats: role of Foxp3 + Treg cells. PLoS ONE. 2016;11(11):1–20. 10.1371/journal.pone.0165916 PMC5089557 27802332

[31] El Skhawy N, et al. Immunomodulatory role of Trichinella spiralis-derived antigen on imiquimod-induced psoriasis in mice model. Parasitol Res. 2024;123(11):397. 39592463 10.1007/s00436-024-08415-7

[32] Eissa MM, et al. Unveiling the anti-neoplastic potential of schistosoma mansoni-derived antigen against breast cancer: a pre-clinical study. Eur J Med Res. 2025;30(1):304. 40247360 10.1186/s40001-025-02531-5 PMC12007238

[33] Eissa MM, et al. Immuno-therapeutic potential of schistosoma mansoni and Trichinella spiralis antigens in a murine model of colon cancer. Invest New Drugs. 2019;37(1):47–56. 29808307 10.1007/s10637-018-0609-6

[34] Zahavi D, Weiner L. Monoclonal antibodies in cancer therapy. Antibodies. 2020;9(3):1–20. 32698317 10.3390/antib9030034 PMC7551545

[35] McGrath JC, Lilley E. Implementing guidelines on reporting research using animals (ARRIVE etc.): new requirements for publication in BJP. Br J Pharmacol. 2015;172(13):3189–93. 25964986 10.1111/bph.12955 PMC4500358

[36] World Medical Association. Declaration of Helsinki: Medical research involving human participants. 2024.

[37] Pellegrino J, Katz N. Experimental chemotherapy of schistosomiasis mansoni. Adv Parasitol. 1968;6:233–90. 4978052 10.1016/s0065-308x(08)60475-3

[38] Pica-Mattoccia L, Cioli D. Sex-and stage-related sensitivity of schistosoma mansoni to in vivo and in vitro praziquantel treatment. Int J Parasitol. 2004;34(4):527–33. 15013742 10.1016/j.ijpara.2003.12.003

[39] Wassom DL, Dougherty DA, Dick TA. Trichinella spiralis infections of inbred mice: immunologically specific responses induced by different Trichinella isolates. J Parasitol. 1988: 283–7. 3357119

[40] McLeod R, et al. Subcutaneous and intestinal vaccination with tachyzoites of Toxoplasma gondii and acquisition of immunity to peroral and congenital Toxoplasma challenge. J Immunol. 1988;140(5):1632–7. 3346545

[41] Fadel EF, et al. Serological and molecular detection of Toxoplasma gondii among cancer patients in Sohag, upper egypt: a case-control study. Sci Rep. 2025;15(1):5236. 39939648 10.1038/s41598-025-88680-3 PMC11822034

[42] Eissa MM, et al. Molecular mimicry between parasites and cancer: a novel approach for developing cancer vaccines and therapeutic antibodies. Cancer Immunol Immunother. 2025;74(7):212. 40402283 10.1007/s00262-025-04069-1 PMC12098237

[43] Rajkapoor B, et al. Antitumor and cytotoxic effects of phyllanthus polyphyllus on Ehrlich Ascites carcinoma and human cancer cell lines. Biosci Biotechnol Biochem. 2007;71(9):2177–83. 17827693 10.1271/bbb.70149

[44] Figueroa D, Asaduzzaman M, Young F. Effect of chemotherapeutics and tocopherols on MCF-7 breast adenocarcinoma and KGN ovarian carcinoma cell lines in vitro. Biomed Res Int. 2019;2019:1–13. 10.1155/2019/6146972 PMC6350544 30766885

[45] Karadayi S, et al. Does hydatid disease have protective effects against lung cancer? Mol Biol Rep. 2013;40:4701–4. 23645038 10.1007/s11033-013-2565-8

[46] Ding J, et al. Excretory-secretory product of Trichinella spiralis inhibits tumor cell growth by regulating the immune response and inducing apoptosis. Acta Trop. 2022;225:106172. 34627760 10.1016/j.actatropica.2021.106172

[47] Bahadory S, et al. In vitro anti-gastrointestinal cancer activity of Toxocara canis-derived peptide: analyzing the expression level of factors related to cell proliferation and tumor growth. Front Pharmacol. 2022;13(878724):1–10. 10.3389/fphar.2022.878724 PMC9530354 36204226

[48] Eissa MM, et al. Evaluation of cytotoxic activity of live Toxoplasma gondii tachyzoites and Toxoplasma antigen on MCF-7 human breast cancer cell line. EUREKA: Life Sci. 2022;2:45–50.

[49] Saravanan BC, et al. A rapid MTT colorimetric assay to assess the proliferative index of two Indian strains of theileria annulata. Vet Parasitol. 2003;113(3–4):211–6. 12719135 10.1016/s0304-4017(03)00062-1

[50] Abdel-Latif M, Sakran T. Detection for cross-reactive proteins in filarial worm setaria equina, MCF-7 human breast cancer, and Huh-7 hepatoma cells. J Immunoass Immunochem. 2016;37(6):572–84. 10.1080/15321819.2016.1179644 27093573

[51] Abdel-Latif M, et al. Immunomodulatory effect of diethylcarbamazine citrate plus filarial excretory–secretory product on rat hepatocarcinogenesis. Int Immunopharmacol. 2015;24(2):173–81. 25499729 10.1016/j.intimp.2014.12.004

[52] Jiang P, et al. Identification of a schistosoma Japonicum MicroRNA that suppresses hepatoma cell growth and migration by targeting host FZD4 gene. Front Cell Infect Microbiol. 2022;12:31. 10.3389/fcimb.2022.786543 PMC8842725 35174106

[53] Shakibapour M, et al. Anti-cancer immunoprotective effects of immunization with hydatid cyst wall antigens in a non-immunogenic and metastatic triple-negative murine mammary carcinoma model. Int Immunopharmacol. 2021;99:107955. 34247052 10.1016/j.intimp.2021.107955

[54] Yue T-T, et al. Anti-osteosarcoma effect of antiserum against cross antigen TPD52 between osteosarcoma and Trichinella spiralis. Parasites Vectors. 2021;14:1–13. 34565443 10.1186/s13071-021-05008-6 PMC8474799

[55] Seyedeh MS, et al. Low titer of antibody against Toxoplasma gondii May be related to resistant to cancer. J Cancer Res Ther. 2015;11(2):305–7. 26148590 10.4103/0973-1482.144638

[56] Salim OE, et al. Colorectal carcinoma associated with schistosomiasis: a possible causal relationship. World J Surg Oncol. 2010;8:1–6. 20704754 10.1186/1477-7819-8-68 PMC2928231

[57] Hu C, et al. A schistosoma Japonicum MicroRNA exerts antitumor effects through Inhibition of both cell migration and angiogenesis by targeting PGAM1. Front Oncol. 2021;11:652395. 34221971 10.3389/fonc.2021.652395 PMC8242254

[58] Yang F, et al. A recombined protein (rSj16) derived from schistosoma Japonicum induces cell cycle arrest and apoptosis of murine myeloid leukemia cells. Parasitol Res. 2013;112:1261–72. 23319265 10.1007/s00436-012-3260-8

[59] Pekkle Lam HY et al. Schistosoma mansoni soluble egg antigen suppresses colorectal cancer growth in vitro and in vivo. J Microbiol Immunol Infect, 2024. 10.1016/j.jmii.2024.11.009 39653602

[60] Loures MA, Andrade A, Peralta JM. Local response in mouse tumor treated with schistosoma mansoni antigen. Mem Inst Oswaldo Cruz, 1991: pp. 127–8. 10.1590/s0074-02761991000700027 1668995

[61] Pereira FEL, Raso P, Coelho PMZ. Evolution of sarcoma 180 (ascitic tumor) in mice infected with schistosoma mansoni. Rev Soc Bras Med Trop. 1986;19:39–42. 3120251 10.1590/s0037-86821986000100009

[62] Cánovas D, Bird N. Human AB serum as an alternative to fetal bovine serum for endothelial and cancer cell culture. ALTEX-ALTERN. 2012;29(4):426–8. 10.14573/altex.2012.4.426 23138512

[63] Heger JI, et al. Human serum alters cell culture behavior and improves spheroid formation in comparison to fetal bovine serum. Exp Cell Res. 2018;365(1):57–65. 29476836 10.1016/j.yexcr.2018.02.017

[64] Li X, et al. Effect of Trichinella on growth of human colorectal carcinoma HCT-8 cells in BALB/c mice. Chin J Biol. 2008;4:285–7.

[65] Apanasevich V, Britov V. Antitumor cross-resistance of trichinosis. Vopr Onkol. 2002;48(2):223–6. 12227073

[66] Weatherly NF. Increased survival of Swiss mice given sublethal infections of Trichinella spiralis. J Parasitol, 1970: pp. 748–52. 5460510

[67] Gong P et al. Observation of anti-tumer effect of Trichinella spirialis in mice on A549 lung cancer cell. Pathogn Biol, 2008: pp. 200–2.

[68] Yue T, et al. Antitumor effect of invasive Lactobacillus plantarum delivering associated antigen gene sHSP between Trichinella spiralis and Lewis lung cancer cells. Int Immunopharmacol. 2023;115:1–12. 10.1016/j.intimp.2023.109708 36638662

[69] Seo S-H, et al. PTEN/AKT signaling pathway related to hTERT downregulation and telomere shortening induced in Toxoplasma GRA16-expressing colorectal cancer cells. Biomed Pharmacother. 2022;153:113366. 35810694 10.1016/j.biopha.2022.113366

[70] Kim J-S, et al. Toxoplasma gondii GRA8-derived peptide immunotherapy improves tumor targeting of colorectal cancer. Oncotarget. 2020;11(1):62. 32002124 10.18632/oncotarget.27417 PMC6967779

[71] Zhu Y-C, et al. Synergy between Toxoplasma gondii type I ∆GRA17 immunotherapy and PD-L1 checkpoint Inhibition triggers the regression of targeted and distal tumors. J Immunother Cancer. 2021;9(11):1–19. 10.1136/jitc-2021-002970 PMC8562526 34725213

[72] Pyo K-H, et al. Prominent IL-12 production and tumor reduction in athymic nude mice after Toxoplasma gondii lysate antigen treatment. Korean J Parasitol. 2014;52(6):605. 25548411 10.3347/kjp.2014.52.6.605 PMC4277022

[73] Pyo K-H, et al. Immune adjuvant effect of a Toxoplasma gondii profilin-like protein in autologous whole-tumor-cell vaccination in mice. Oncotarget. 2016;7(45):74107. 27687589 10.18632/oncotarget.12316 PMC5342039

[74] Mani R, et al. A novel protozoa parasite-derived protein adjuvant is effective in immunization with cancer cells to activate the cancer-specific protective immunity and inhibit the cancer growth in a murine model of colorectal cancer. Cells. 2024;13(2):111. 38247803 10.3390/cells13020111 PMC10814441

[75] Lu J, et al. Exosomes derived from dendritic cells infected with Toxoplasma gondii show antitumoral activity in a mouse model of colorectal cancer. Front Oncol. 2022;12:899737. 35600363 10.3389/fonc.2022.899737 PMC9114749

[76] Zhu S, et al. Anti-tumoral effect and action mechanism of exosomes derived from Toxoplasma gondii-infected dendritic cells in mice colorectal cancer. Front Oncol. 2022;12:1–13. 10.3389/fonc.2022.870528 PMC9118538 35600340

[77] Arul M, Roslani AC, Cheah SH. Heterogeneity in cancer cells: variation in drug response in different primary and secondary colorectal cancer cell lines in vitro. Vitro Cell Dev Biol Anim. 2017;53(5):435–47. 10.1007/s11626-016-0126-x 28120247

[78] Motavallihaghi S, et al. In vitro anticancer activity of hydatid cyst fluid on colon cancer cell line (C26). Egypt J Med Hum Genet. 2023;24(1):15.

[79] Berriel E, et al. Antitumor activity of human hydatid cyst fluid in a murine model of colon cancer. Sci World J. 2013;2013(2013):1–7. 10.1155/2013/230176 PMC3759250 24023528

[80] Rostamirad S, et al. Inhibition of mouse colon cancer growth following immunotherapy with a fraction of hydatid cyst fluid. Exp Parasitol. 2023;249:108501. 36931383 10.1016/j.exppara.2023.108501

[81] Jacobs B-A, Prince S, Smith KA. Gastrointestinal nematode-derived antigens alter colorectal cancer cell proliferation and migration through regulation of cell cycle and epithelial-mesenchymal transition proteins. Int J Mol Sci. 2020;21(21):7845. 33105843 10.3390/ijms21217845 PMC7660063

[82] Ding Y, et al. The plasmodium circumsporozoite protein, a novel NF-κB inhibitor, suppresses the growth of SW480. Pathol Oncol Res. 2012;18:895–902. 22678765 10.1007/s12253-012-9519-7

[83] Yao X, et al. Plasmodium infection suppresses colon cancer growth by inhibiting proliferation and promoting apoptosis associated with disrupting mitochondrial biogenesis and mitophagy in mice. Parasit Vectors. 2022;15(1):1–12. 35668501 10.1186/s13071-022-05291-x PMC9169289

[84] Oliveira E, et al. Chronic trypanosoma Cruzi infection associated with low incidence of 1, 2-dimethylhydrazine-induced colon cancer in rats. Carcinogenesis. 2001;22(5):737–40. 11323392 10.1093/carcin/22.5.737

[85] Ubillos L, et al. Trypanosoma Cruzi extracts elicit protective immune response against chemically induced colon and mammary cancers. Int J Cancer. 2016;138(7):1719–31. 26519949 10.1002/ijc.29910

[86] Huang H, et al. Antitumour metastasis and the antiangiogenic and antitumour effects of a Eimeria stiedae soluble protein. Parasite Immunol. 2021;43(6):e12825. 33507547 10.1111/pim.12825

[87] León-Cabrera S, et al. Extraintestinal helminth infection reduces the development of colitis-associated tumorigenesis. Int J Biol Sci. 2014;10(9):948. 25210492 10.7150/ijbs.9033 PMC4159685

[88] Hu C, et al. Schistosoma Japonicum MiRNA-7-5p inhibits the growth and migration of hepatoma cells via cross-species regulation of S-phase kinase-associated protein 2. Front Oncol. 2019;9:175. 30967999 10.3389/fonc.2019.00175 PMC6443022

[89] Hu S, et al. Anti-inflammatory protein of schistosoma Japonicum directs the differentiation of the wehi‐3b Jcs cells and mouse bone marrow cells to macrophages. BioMed Res Int. 2010;2010(1):867368. 10.1155/2010/867368 PMC2831513 20204135

[90] Lin Y, et al. Cross-species suppression of hepatoma cell growth and migration by a schistosoma Japonicum MicroRNA. Mol Ther Nucleic Acids. 2019;18:400–12. 31655260 10.1016/j.omtn.2019.09.006 PMC6831938

[91] Palumbo E. Association between schistosomiasis and cancer, a review. Infect Dis Clin Pract. 2007;15(3):145–8.

[92] Wahib A, et al. Cell mediated immune response in chronic liver diseases: schistosomal, viral and neoplastic. J Egypt Soc Parasitol. 1998;28(3):929–39. 9914713

[93] El-Tonsy MM, et al. Schistosoma mansoni infection: is it a risk factor for development of hepatocellular carcinoma? Acta Trop. 2013;128(3):542–7. 23932944 10.1016/j.actatropica.2013.07.024

[94] Roderfeld M, et al. Schistosoma mansoni egg-secreted antigens activate hepatocellular carcinoma-associated transcription factors c-Jun and STAT3 in hamster and human hepatocytes. Hepatology. 2020;72(2):626–41. 30053321 10.1002/hep.30192 PMC7496692

[95] Wang X, et al. Trichinella spiralis—A potential anti-tumor agent. Vet Parasitol. 2009;159(3–4):249–52. 19041180 10.1016/j.vetpar.2008.10.052

[96] Ruenchit P, et al. Peptide of Trichinella spiralis infective larval extract that harnesses growth of human hepatoma cells. Front Cell Infect Microbiol. 2022;12:882608. 35558100 10.3389/fcimb.2022.882608 PMC9086976

[97] Zhang Y, et al. Anti-tumoral effect of Trichinella spirialis on Hepa 1–6 hepatoma carcinoma cell in the C57BL/6 mice. J Pathogen Biology. 2009;4(1):24–6.

[98] Elhasawy FA, et al. The apoptotic effect of Trichinella spiralis infection against experimentally induced hepatocellular carcinoma. Asian Pac J Cancer Prev. 2021;22(3):935. 33773560 10.31557/APJCP.2021.22.3.935 PMC8286675

[99] Sadr S, et al. Trichinella spiralis as a potential antitumor agent: an update. World’s Vet J, 2023(1): pp. 65–74.

[100] Luo J, et al. Study on the mitochondrial apoptosis pathways of small cell lung cancer H446 cells induced by Trichinella spiralis muscle larvae esps. Parasitology. 2017;144(6):793–800. 28073393 10.1017/S0031182016002535

[101] Wang H, et al. Transcriptome profiling of A549 non-small cell lung cancer cells in response to Trichinella spiralis muscle larvae excretory/secretory products. Front Vet Sci. 2023;10:1–11. 10.3389/fvets.2023.1208538 PMC10433203 37601754

[102] Wu H, et al. Trichinella spiralis muscle larvae excretory/secretory products trigger apoptosis and S-phase arrest of the non-small-cell lung cancer line A549. Exp Parasitol. 2020;218:1–6. 10.1016/j.exppara.2020.107983 32861680

[103] Meerovitch E, Bomford R. Macrophage potentiation by Trichinella spiralis. Ann Trop Med Parasit. 1977;71(2):245–7. 869616 10.1080/00034983.1977.11687187

[104] Piaggi S, et al. Glutathione-S-transferase Omega 1 and nurse cell formation during experimental Trichinella infection. Vet Parasitol. 2021;297:109114. 32386865 10.1016/j.vetpar.2020.109114

[105] Wing E, Krahenbuhl J, Remington J. Studies of macrophage function during Trichinella spiralis infection in mice. Immunology. 1979;36(3):1–11. 437839 PMC1457586

[106] Vasilev S, et al. Experimental immunology necrosis and apoptosis in Trichinella spiralis-mediated tumour reduction. Cent Eur J Immunol. 2015;40(1):42–53. 26155183 10.5114/ceji.2015.50832 PMC4472539

[107] Tsocheva-Gaytandzhieva N, et al. Antiproliferative activity against tumour cells of biologically active substances isolated from livers of healthy and Trichinella spiralis infected rats. C R Acad Bulg Sci. 2016;69(11):1443–8.

[108] Deng B, et al. Identification of the differentially expressed genes in SP2/0 myeloma cells from Balb/c mice infected with Trichinella spiralis. Vet Parasitol. 2013;194(2–4):179–82. 23473833 10.1016/j.vetpar.2013.01.050

[109] Lubiniecki A, Cypess R. Quantitative study of the effect of previous Trichinella spiralis infection on sarcoma 180 ascitic tumor formation in mice. Tropenmed Parasitol. 1975;26(3):329–34. 1189027

[110] Molinari J, Carrick L Jr, Lubiniecki A. Influence of Trichinella spiralis infection on development of sarcoma-180 Ascites tumors. Tropenmed Parasitol. 1979;30(4):429–33. 538817

[111] Molinari J, Ebersole J. Antineoplastic effects of long-term Trichinella spiralis infection on B-16 melanoma. Int Arch Allergy Appl Immunol. 1977;55(1–6):444–8. 591108 10.1159/000231956

[112] Pocock D, Meerovitch E. The anti-neoplastic effect of trichinellosis in a syngeneic murine model. Parasitology. 1982;84(3):463–73. 7099711 10.1017/s0031182000052768

[113] Kang Y-J, et al. Trichinella spiralis infection reduces tumor growth and metastasis of B16-F10 melanoma cells. Vet Parasitol. 2013;196(1–2):106–13. 23499484 10.1016/j.vetpar.2013.02.021

[114] Liu J, et al. Observation of Trichinella on C6 glioma in BALB/c mice. J Apoplexy Nerv Dis. 2008;6:722–4.

[115] Wang G, Gao M. Influence of Toxoplasma gondii on in-vitro proliferation and apoptosis of hepatoma carcinoma H7402 cell. Asian Pac J Trop Med. 2016;9(1):63–6. 26851789 10.1016/j.apjtm.2015.12.013

[116] Kim SG, et al. Increase in the nuclear localization of PTEN by the Toxoplasma GRA16 protein and subsequent induction of p53-dependent apoptosis and anticancer effect. J Cell Mol Med. 2019;23(5):3234–45. 30834688 10.1111/jcmm.14207 PMC6484329

[117] Li Y, et al. Macrophages polarized by expression of ToxoGRA15II inhibit growth of hepatic carcinoma. Front Immunol. 2017;8:137. 28243242 10.3389/fimmu.2017.00137 PMC5303709

[118] Abdoli A, et al. Screening of toxoplasmosis in cancer patients: a concern. Trop Doct. 2019;49(1):31–4. 30270766 10.1177/0049475518801618

[119] Cong W, et al. Toxoplasma gondii infection in cancer patients: prevalence, risk factors, genotypes and association with clinical diagnosis. Cancer Lett. 2015;359(2):307–13. 25641340 10.1016/j.canlet.2015.01.036

[120] Daher D, et al. Comprehensive overview of Toxoplasma gondii-induced and associated diseases. Pathogens. 2021;10(11):1351. 34832507 10.3390/pathogens10111351 PMC8625914

[121] Wang Z-D, et al. Toxoplasma gondii infection in immunocompromised patients: a systematic review and meta-analysis. Front Microbiol. 2017;8:1–12. 28337191 10.3389/fmicb.2017.00389 PMC5343064

[122] Ahmed DF, Saheb EJ. The association of Toxoplasma gondii infection in breast and colorectal cancer patients. IJCOCR. 2017;2(4):86–92.

[123] Ali MI, et al. Toxoplasma gondii in cancer patients receiving chemotherapy: Seroprevalence and interferon gamma level. J Parasit Dis. 2019;43(3):464–71. 31406412 10.1007/s12639-019-01111-9 PMC6667530

[124] Yu Y, et al. Increased risk of Toxoplasma gondii infection in patients with colorectal cancer in Eastern china: seroprevalence, risk factors, and a case–control study. Biomed Res Int. 2020;2020(1):2539482. 33083457 10.1155/2020/2539482 PMC7563061

[125] Klastersky J, Aoun M. Opportunistic infections in patients with cancer. Ann Oncol. 2004;15:iv329–35. 15477331 10.1093/annonc/mdh947

[126] El Skhawy N, Eissa MM. Shedding light on a mysterious link between Toxoplasma gondii and cancer: A review. Exp Parasitol. 2023;250:108544. 37149210 10.1016/j.exppara.2023.108544

[127] Luo Q, et al. Effect of culture supernatant of Toxoplasma gondii on the proliferation and apoptosis of BGC-823 cells. Zhongguo Ji Sheng Chong Xue Yu Ji Sheng Chong Bing Za Zhi ‘Chinese. J Parasitol Parasitic Diseases’. 2014;32(2):123–7. 25065211

[128] Xin W, et al. Impact of Toxoplasma gondii on the proliferation and apoptosis of tumor cell lines. Zhongguo Ji Sheng Chong Xue Yu Ji Sheng Chong Bing Za Zhi. Chin J Parasitol Parasitic Dis. 2012;30(2):17. 22908820

[129] Şahar EA, et al. Toxoplasma gondii destroys Her2/Neu-expressing mammary cancer cells in vitro using a continuous feed medium approach. J Infect Dev Ctries. 2020;14(10):1204–9. 33175718 10.3855/jidc.12820

[130] Miyahara K, et al. Therapeutic effects of Toxoplasma lysate antigen on 20-methylcholanthrene-induced BALB/c mouse tumors. J Vet Med Sci. 1992;54(1):7–12. 1558892 10.1292/jvms.54.7

[131] Shirzad H, et al. Toxoplasma gondii but not leishmania major or Trichomonas vaginalis decreases cell proliferation and increases cell death on fibrosarcoma cancer cells in culture medium. World J Vaccines. 2012;2(2):105–8.

[132] Elikaei A, Vazini H, Javani F. Anticancer effects of parasite extracts of leishmaniasis and Toxoplasma on resistant cell line (A2780-CP) and sensitive (A2780) to cisplatin. Appl Biol. 2018;31(2):5–22.

[133] Payne SN, et al. Novel murine pancreatic tumor model demonstrates immunotherapeutic control of tumor progression by a Toxoplasma gondii protein. Infect Immun. 2021;89(12). 10.1128/IAI.00508-21 PMC8594601 34543124

[134] Sanders KL, Fox BA, Bzik DJ. Attenuated Toxoplasma gondii stimulates immunity to pancreatic cancer by manipulation of myeloid cell populations. Cancer Immunol Res. 2015;3(8):891–901. 25804437 10.1158/2326-6066.CIR-14-0235 PMC4526316

[135] Sanders KL, Fox BA, Bzik DJ. Attenuated Toxoplasma gondii therapy of disseminated pancreatic cancer generates long-lasting immunity to pancreatic cancer. Oncoimmunology. 2016;5(4):e1104447. 27141388 10.1080/2162402X.2015.1104447 PMC4839330

[136] Hibbs JB Jr, Lambert LH Jr, Remington JS. Resistance to murine tumors conferred by chronic infection with intracellular protozoa, Toxoplasma gondii and besnoitia Jellisoni. J Infect Dis. 1971;124(6):587–92. 5127071 10.1093/infdis/124.6.587

[137] Xu L-Q, et al. A uracil auxotroph Toxoplasma gondii exerting Immunomodulation to inhibit breast cancer growth and metastasis. Parasit Vectors. 2021;14(1):601. 34895326 10.1186/s13071-021-05032-6 PMC8665513

[138] Kim BK, et al. Anti-tumor effects of Toxoplasma gondii and antigen-pulsed dendritic cells in mice bearing breast cancer. Parasites Hosts Dis. 2025;63(1):37. 40045679 10.3347/PHD.24082 PMC11895089

[139] Kim J-O, et al. Inhibition of Lewis lung carcinoma growth by Toxoplasma gondii through induction of Th1 immune responses and Inhibition of angiogenesis. J Korean Med Sci. 2007;22(Suppl):S38. 17923753 10.3346/jkms.2007.22.S.S38 PMC2694397

[140] Yu-Meng J, et al. Inhibition of Toxoplasma gondii excretory-secretory antigens on growth of murine Lewis lung carcinoma. Chin J Schistosomiasis Control. 2019;31(4):400. 10.16250/j.32.1374.2018269 31612675

[141] Darani HY, et al. Effects of Toxoplasma gondii and Toxocara canis antigens on WEHI-164 fibrosarcoma growth in a mouse model. Korean J Parasitol. 2009;47(2):175. 19488426 10.3347/kjp.2009.47.2.175 PMC2688801

[142] Amari A et al. Effects of dendritic cell vaccine activated with protein components of Toxoplasma gondii on tumor specific CD8 + T-cells. Tehran Univ Med J. 2009;67(9).

[143] Motamedi M, et al. Improvement of a dendritic cell-based therapeutic cancer vaccine with components of Toxoplasma gondii. Clin Vaccine Immunol. 2009;16(10):1393–8. 19656994 10.1128/CVI.00199-09 PMC2756855

[144] Pyo K-H, et al. Suppressed CD31 expression in sarcoma-180 tumors after injection with Toxoplasma gondii lysate antigen in BALB/c mice. Korean J Parasitol. 2010;48(2):171. 20585536 10.3347/kjp.2010.48.2.171 PMC2892575

[145] Boghozian R, et al. Identification of Toxoplasma gondii protein fractions induce immune response against melanoma in mice. Apmis. 2015;123(9):800–9. 26152792 10.1111/apm.12420

[146] Hunter CA, et al. Cutting edge: systemic Inhibition of angiogenesis underlies resistance to tumors during acute toxoplasmosis. J Immunol. 2001;166(10):5878–81. 11342601 10.4049/jimmunol.166.10.5878

[147] Jiao YuMeng JY, et al. Effects of excreted/secreted antigens of Toxoplasma gondii on CD4 + CD25 + Foxp3 + T cells and NK cells of melanoma-bearing mice. Zhongguo Xue Xi Chong Bing Fang Zhi Za Zhi. 2011;23(3):301–6. 22164498

[148] Fox BA, et al. Secretion of Rhoptry and dense granule effector proteins by nonreplicating Toxoplasma gondii uracil auxotrophs controls the development of antitumor immunity. PLoS Genet. 2016;12(7):e1006189. 27447180 10.1371/journal.pgen.1006189 PMC4957766

[149] Conley FK, Remington JS. Nonspecific Inhibition of tumor growth in the central nervous system: observations of intracerebral Ependymoblastoma in mice with chronic Toxoplasma infection. J Natl Cancer Inst. 1977;59(3):963–73. 894752 10.1093/jnci/59.3.963

[150] Lantier L, et al. Neospora caninum: a new class of biopharmaceuticals in the therapeutic arsenal against cancer. J Immunother Cancer. 2020;8(2):e001242. 33257408 10.1136/jitc-2020-001242 PMC7705568

[151] Darani HY, Sharafi SM. Effect of hydatid cyst antigens polyspecific antisera on breast cancer cells (4T1) growth in cell culture medium. Int J Env Health Eng. 2022;11(1):15.

[152] Eligio García L, et al. Trypanosoma Cruzi antigenic proteins shared with acute lymphoblastic leukemia and neuroblastoma. Pharmaceuticals. 2022;15(11):1421. 36422551 10.3390/ph15111421 PMC9693088

[153] Maravelez Acosta VA, et al. Association of neuroblastoma (nb) SH-SY5Y cells with antibodies of parasitic origin (anti-Acanthamoeba and anti-Toxocara canis). Int J Mol Sci. 2024;25(24):13577. 39769340 10.3390/ijms252413577 PMC11678856

[154] Freire T, et al. Trypanosoma cruzi-derived molecules induce anti-tumour protection by favouring both innate and adaptive immune responses. Int J Mol Sci. 2022;23(23):15032. 36499361 10.3390/ijms232315032 PMC9739173

[155] Berriel E, et al. Human hydatid cyst fluid-induced therapeutic anti-cancer immune responses via NK1. 1 + cell activation in mice. Cancer Immunol Immunother. 2021;70(12):3617–27. 33944981 10.1007/s00262-021-02948-x PMC10992520

[156] Mohamadi F, et al. Anti-Toxoplasma gondii antibodies attach to mouse cancer cell lines but not normal mouse lymphocytes. Biomed Rep. 2019;10(3):183–8. 30906547 10.3892/br.2019.1186 PMC6403471

[157] Angsubhakorn S, et al. Reducing effects of rodent malaria on hepatic carcinogenesis induced by dietary aflatoxin B1. Int J Cancer. 1988;41(1):69–73. 3121525 10.1002/ijc.2910410114

[158] Wang B, et al. Plasmodium infection inhibits tumor angiogenesis through effects on tumor-associated macrophages in a murine implanted hepatoma model. Cell Commun Signal. 2020;18:1–17. 32972437 10.1186/s12964-020-00570-5 PMC7513281

[159] Lu Y, et al. Immunomodulatory action of excretory-secretory products of Angiostrongylus cantonensis in a mouse tumour model. Parasitol Res. 2020;119:3705–18. 32901341 10.1007/s00436-020-06872-4

[160] Abdel-Latif M, et al. Effect of diethylcarbamazine citrate and setaria equina excretory–secretory material on rat hepatocellular carcinoma. Arch Immunol Ther Exp. 2014;62:511–20. 10.1007/s00005-014-0292-z 24879096

[161] Calabrese EJ. Biphasic dose responses in biology, toxicology and medicine: accounting for their generalizability and quantitative features. Environ Pollut. 2013;182:452–60. 23992683 10.1016/j.envpol.2013.07.046

