# Supplementary material for: Parasites’ immunomodulators: a breakthrough in immunotherapeutics displaying antineoplastic activity against human colorectal and hepatocellular carcinoma cells
Source: Infect Agent Cancer. 2025 Dec 12;21:5. doi: 10.1186/s13027-025-00715-6 (PMC12805742; doi:10.1186/s13027-025-00715-6)
Supplement: Supplementary file 1 — Supplementary Material 1 [file 13027_2025_715_MOESM1_ESM.docx]

**Table (1): HT-29 colorectal cancer cell proliferation and inhibition percentages upon incubation with different concentrations of parasitic antigens for 24 hours,** **compared to cells cultured in media only**

| **Parasitic**  **Antigens’**  **Concentrations** | **Control** | **A*Sm*A** | **A*Ts*A** | **A*Tg*A** | **F** | **p** |
| --- | --- | --- | --- | --- | --- | --- |
| **100 µg/ml**  **% of proliferation** | 100 ± 0 | 76.56^cef^ ± 0.48 | 73.33^cdef^ ± 0.98 | 72.15^cdef^ ±0.79 | 1136.527^*^ | <0.001^*^ |
| **% of inhibition** |  | **23.44** | **26.67** | **27.85** |  |  |
| **p_control_** |  | <0.001^*^ | <0.001^*^ | <0.001^*^ |  |  |
|  |  | p_1_=0.002^*^,p_2_<0.001^*^,p_3_=0.219 | | |  |  |
| **50 µg/ml**  **% of proliferation** | 100 ± 0 | 75.24^cef^ ± 0.21 | 74.57^cdef^ ± 0.55 | 72.45^cdef^ ± 0.81 | 2022.577^*^ | <0.001^*^ |
| **% of inhibition** |  | **24.76** | **25.43** | **27.55** |  |  |
| **p_control_** |  | <0.001^*^ | <0.001^*^ | <0.001^*^ |  |  |
|  |  | p_1_=0.404,p_2_=0.001^*^,p_3_=0.004^*^ | | |  |  |
| **25 µg/ml**  **% of proliferation** | 100 ± 0 | 68.28^abdef^ ± 0.07 | 63.35^abdef^ ± 0.29 | 68.77^abdef^ ± 0.66 | 6389.829^*^ | <0.001^*^ |
| **% of inhibition** |  | **31.72** | **36.65** | **31.23** |  |  |
| **p_control_** |  | <0.001^*^ | <0.001^*^ | <0.001^*^ |  |  |
|  |  | p_1_<0.001^*^,p_2_=0.395,p_3_<0.001^*^ | | |  |  |
| **12.5 µg/ml**  **% of proliferation** | 100 ± 0 | 76.50^cef^ ± 1.10 | 70.88^abcef^ ± 0.91 | 90.82^abc^ ± 0.37 | 972.921^*^ | <0.001^*^ |
| **% of inhibition** |  | **23.5** | **29.12** | **9.18** |  |  |
| **p_control_** |  | <0.001^*^ | <0.001^*^ | <0.001^*^ |  |  |
|  |  | p_1_<0.001^*^,p_2_<0.001^*^,p_3_<0.001^*^ | | |  |  |
| **6.25 µg/ml**  **% of proliferation** | 100 ± 0 | 78.33^abcd^ ± 0.15 | 80.31^abcd^ ± 0.65 | 90.83^abc^ ± 0.65 | 1395.638^*^ | <0.001^*^ |
| **% of inhibition** |  | **21.67** | **19.69** | **9.17** |  |  |
| **p_control_** |  | <0.001^*^ | <0.001^*^ | <0.001^*^ |  |  |
|  |  | p_1_=0.004^*^,p_2_<0.001^*^,p_3_<0.001^*^ | | |  |  |
| **3.125 µg/ml**  **% of proliferation** | 100 ± 0 | 78.52^abcd^ ± 0.30 | 80.47^abcd^ ± 1.12 | 92.09^abc^ ± 0.71 | 670.311^*^ | <0.001^*^ |
| **% of inhibition** |  | **21.48** | **19.53** | **7.91** |  |  |
| **p_control_** |  | <0.001^*^ | <0.001^*^ | <0.001^*^ |  |  |
|  |  | p_1_=0.032^*^,p_2_<0.001^*^,p_3_<0.001^*^ | | |  |  |
| **F** | – | 159.496^*^ | 192.472^*^ | 799.943^*^ |  |  |
| **p_4_** | – | <0.001^*^ | <0.001^*^ | <0.001^*^ |  |  |

A*Sm*A: Autoclaved *Schistosoma mansoni* antigen, A*Ts*A: Autoclaved *Trichinella spiralis* antigen and A*Tg*A: Autoclaved *Toxoplasma gondii* antigen

Data was expressed using Mean ± Standard deviation (SD)

F: F for One-way ANOVA test, Pairwise comparison between each two groups was done using Post Hoc Test (Tukey)

P_control_: p value for comparing between the control and other groups

p_1_: p value for comparing between the A*Sm*A and A*Ts*A

p_2_: p value for comparing between the A*Sm*A and A*Tg*A

p_3_: p value for comparing between the A*Ts*A and A*Tg*A

p_4_: p value for comparing between the different studied concentrations of the same antigen

*: Statistically significant at p ≤ 0.05

a: Significant with **100 µg/ml**

b: Significant with **50 µg/ml**

c: Significant with **25 µg/ml**

d: Significant with **12.5 µg/ml**

e: Significant with **6.25 µg/ml**

f: Significant with **3.125 µg/ml**

**Table (2): HT-29 colorectal cancer cell proliferation and inhibition percentages upon incubation with different concentrations of sera positive for parasitic IgG antibodies for 24 hours, compared** **to cells cultured in media only and sera negative for parasitic IgG, negative controls**

| **Serum ^+/-^ for parastic IgG** | **Control** | **Sera negative for parasitic IgG** | ***S. mansoni* IgG^+^sera** | ***T. spiralis* IgG^+^sera** | ***T. gondii***  **IgG^+^sera** | **F** | **p** |
| --- | --- | --- | --- | --- | --- | --- | --- |
| **40%**  **% of proliferation** | 100 ± 0 | 126.2^bcd^ ± 1.74 | 79.29^cde^ ± 0.16 | 63.23^cde^ ± 1.12 | 62.56^bcde^ ± 2.39 | 1093.879^*^ | <0.001^*^ |
| **% of inhibition** |  |  | **37.17** | **49.90** | **50.43** |  |  |
| **p_control_** |  | <0.001^*^ | <0.001^*^ | <0.001^*^ | <0.001^*^ |  |  |
| **p_0_** |  |  | <0.001^*^ | <0.001^*^ | <0.001^*^ |  |  |
|  |  |  | p_1_<0.001^*^,p_2_<0.001^*^,p_3_=0.975 | | |  |  |
| **20%**  **% of proliferation** | 100 ± 0 | 108.4^a^ ± 1.72 | 81.48^cde^ ± 3.76 | 65.88^cde^ ± 0.53 | 72.29^acde^ ± 1.78 | 239.615^*^ | <0.001^*^ |
| **% of inhibition** |  |  | **24.83** | **39.23** | **33.31** |  |  |
| **p_control_** |  | 0.003^*^ | <0.001^*^ | <0.001^*^ | <0.001^*^ |  |  |
| **p_0_** |  |  | <0.001^*^ | <0.001^*^ | <0.001^*^ |  |  |
|  |  |  | p_1_<0.001^*^,p_2_=0.002^*^,p_3_=0.020^*^ | | |  |  |
| **10%**  **% of proliferation** | 100 ± 0 | 104.9^a^ ± 5.65 | 89.73^ab^ ± 0.25 | 70.41^abde^ ± 1.31 | 87.55^abe^ ± 1.22 | 75.629^*^ | <0.001^*^ |
| **% of inhibition** |  |  | **14.46** | **32.88** | **16.54** |  |  |
| **p_control_** |  | 0.239 | 0.005^*^ | <0.001^*^ | 0.001^*^ |  |  |
| **p_0_** |  |  | <0.001^*^ | <0.001^*^ | <0.001^*^ |  |  |
|  |  |  | p_1_<0.001^*^,p_2_=0.847,p_3_<0.001^*^ | | |  |  |
| **5%**  **% of proliferation** | 100 ± 0 | 102.5^a^ ± 3.51 | 89.36^ab^ ± 0.29 | 74.35^abce^ ± 1.19 | 90.37^abe^ ± 2.69 | 87.811^*^ | <0.001^*^ |
| **% of inhibition** |  |  | **12.82** | **27.46** | **11.83** |  |  |
| **p_control_** |  | 0.596 | 0.001^*^ | <0.001^*^ | 0.001^*^ |  |  |
| **p_0_** |  |  | <0.001^*^ | <0.001^*^ | <0.001^*^ |  |  |
|  |  |  | p_1_<0.001^*^,p_2_=0.972,p_3_<0.001^*^ | | |  |  |
| **2.5%**  **% of proliferation** | 100 ± 0 | 100.6^a^ ± 0.34 | 89.36^ab^ ± 1.04 | 81.61^abcd^ ± 0.96 | 114.9^abcd^ ± 2.12 | 363.718^*^ | <0.001^*^ |
| **% of inhibition** |  |  | **11.17** | **18.88** | **-14.21** |  |  |
| **p_control_** |  | 0.957 | <0.001^*^ | <0.001^*^ | <0.001^*^ |  |  |
| **p_0_** |  |  | <0.001^*^ | <0.001^*^ | <0.001^*^ |  |  |
|  |  |  | p_1_<0.001^*^,p_2_<0.001^*^,p_3_<0.001^*^ | | |  |  |
| **F** | – | 31.735^*^ | 24.852^*^ | 141.688^*^ | 270.982^*^ |  |  |
| **p_4_** | – | <0.001^*^ | <0.001^*^ | <0.001^*^ | <0.001^*^ |  |  |

Data was expressed using Mean ± Standard deviation (SD)

F: F for One-way ANOVA test, Pairwise comparison between each two groups was done using Post Hoc Test (Tukey)

P_control_: p value for comparing between the media-only control and other groups

p_0_: p value for comparing between the sera negative for parasitic IgG and other groups

p_1_: p value for comparing between the *S.mansoni* IgG^+^sera and *T.spiralis* IgG^+^sera

p_2_: p value for comparing between the *S.mansoni* IgG^+^sera and *T. gondii* IgG^+^sera

p_3_: p value for comparing between the *T.spiralis* IgG^+^sera and *T. gondii* IgG^+^sera

p_4_: p value for comparing between the different studied concentrations of the same IgG-positive sera

*: Statistically significant at p ≤ 0.05

a: Significant with **40%**

b: Significant with **20%**

c: Significant with **10%**

d: Significant with **5%**

e: Significant with **2.5%**

**Table (3): HepG2 hepatocellular carcinoma cell proliferation and inhibition percentages upon incubation with different concentrations of parasitic antigens for 24 hours,** **compared to cells cultured in media only**

| **Parasitic Antigens’**  **Concentrations** | **Control** | **A*Sm*A** | **A*Ts*A** | **A*Tg*A** | **F** | | **p** | |  |
| --- | --- | --- | --- | --- | --- | --- | --- | --- | --- |
| **100 µg/ml**  **% of proliferation** | 100 ± 0 | 91.97^def^ ± 2.35 | 61.73^bcdef^ ± 1.96 | 65.32^bcdef^ ± 3.07 | 232.843^*^ | | <0.001^*^ | |  |
| **% of inhibition** |  | **8.03** | **38.27** | **34.68** |  | |  | |  |
| **p_control_** |  | 0.008^*^ | <0.001^*^ | <0.001^*^ |  | |  | |  |
|  |  | p_1_<0.001^*^,p_2_<0.001^*^,p_3_=0.255 | | | |  | |  | |
| **50 µg/ml**  **% of proliferation** | 100 ± 0 | 92.77^def^ ± 1.89 | 66.27^acdef^ ± 1 | 76.83^acdef^ ± 2.32 | 280.877^*^ | | <0.001^*^ | |  |
| **% of inhibition** |  | **7.23** | **33.73** | **23.17** |  | |  | |  |
| **p_control_** |  | 0.002^*^ | <0.001^*^ | <0.001^*^ |  | |  | |  |
|  |  | p_1_<0.001^*^,p_2_<0.001^*^,p_3_<0.001^*^ | | | |  | |  | |
| **25 µg/ml**  **% of proliferation** | 100 ± 0 | 96.30^ef^ ± 0.75 | 71.63^abdef^ ± 2.20 | 85.11^abf^ ± 0.96 | 309.235^*^ | | <0.001^*^ | |  |
| **% of inhibition** |  | **3.7** | **28.37** | **14.89** |  | |  | |  |
| **p_control_** |  | 0.029^*^ | <0.001^*^ | <0.001^*^ |  | |  | |  |
|  |  | p_1_<0.001^*^,p_2_<0.001^*^,p_3_<0.001^*^ | | | |  | |  | |
| **12.5 µg/ml**  **% of proliferation** | 100 ± 0 | 104.1^abf^ ± 5.34 | 78.97^abcef^ ± 1.47 | 85.32^abf^ ± 2.01 | 49.099^*^ | | <0.001^*^ | |  |
| **% of inhibition** |  | **-4.1** | **21.03** | **14.68** |  | |  | |  |
| **p_control_** |  | 0.373 | <0.001^*^ | 0.001^*^ |  | |  | |  |
|  |  | p_1_<0.001^*^,p_2_<0.001^*^,p_3_=0.110 | | | |  | |  | |
| **6.25 µg/ml**  **% of proliferation** | 100 ± 0 | 105.7^abcf^ ± 3.40 | 94.27^abcd^ ± 0.81 | 86.73^abf^ ± 1.06 | 58.933^*^ | | <0.001^*^ | |  |
| **% of inhibition** |  | **-5.7** | **5.73** | **13.27** |  | |  | |  |
| **p_control_** |  | 0.022^*^ | 0.021^*^ | <0.001^*^ |  | |  | |  |
|  |  | p_1_<0.001^*^,p_2_<0.001^*^,p_3_=0.004^*^ | | | |  | |  | |
| **3.125 µg/ml**  **% of proliferation** | 100 ± 0 | 120.9^abcde^ ± 3.05 | 95.53^abcd^ ± 1.43 | 112.2^abcde^ ± 1.92 | 106.371^*^ | | <0.001^*^ | |  |
| **% of inhibition** |  | **-20.9** | **4.47** | **-12.2** |  | |  | |  |
| **p_control_** |  | <0.001^*^ | 0.085 | <0.001^*^ |  | |  | |  |
|  |  | p_1_<0.001^*^,p_2_=0.003^*^,p_3_<0.001^*^ | | | |  | |  | |
| **F** | – | 36.138^*^ | 251.836^*^ | 175.222^*^ |  | |  | |  |
| **p_4_** | – | <0.001^*^ | <0.001^*^ | <0.001^*^ |  | |  | |  |

A*Sm*A: Autoclaved *Schistosoma mansoni* antigen, A*Ts*A: Autoclaved *Trichinella spiralis* antigen and A*Tg*A: Autoclaved *Toxoplasma gondii* antigen

Data was expressed using Mean ± Standard deviation (SD)

F: F for One-way ANOVA test, Pairwise comparison between each two groups was done using Post Hoc Test (Tukey)

P_control_: p value for comparing between the control and other groups

p_1_: p value for comparing between the A*Sm*A and A*Ts*A

p_2_: p value for comparing between the A*Sm*A and A*Tg*A

p_3_: p value for comparing between the A*Ts*A and A*Tg*A

p_4_: p value for comparing between the different studied concentrations of the same antigen

*: Statistically significant at p ≤ 0.05

a: Significant with **100 µg/ml**

b: Significant with **50 µg/ml**

c: Significant with **25 µg/ml**

d: Significant with **12.5 µg/ml**

e: Significant with **6.25 µg/ml**

f: Significant with **3.125 µg/ml**

**Table (4): HepG2 hepatocellular carcinoma cell proliferation and inhibition percentages upon incubation with different concentrations of sera positive for parasitic IgG antibodies for 24 hours,** **compared to cells cultured in media only and sera negative for parasitic IgG, negative controls**

| **Serum ^+/-^ for parasitic IgG** | **Control** | **Sera negative for parasitic IgG** | ***S.mansoni* IgG^+^sera** | ***T.spiralis* IgG^+^sera** | ***T. gondii***  **IgG^+^sera** | **F** | **p** |
| --- | --- | --- | --- | --- | --- | --- | --- |
| **40%**  **% of proliferation** | 100 ± 0 | 119.8^bcde^ ± 2.35 | 114.4^bcde^ ± 1.68 | 62^bcde^ ± 2.75 | 69.51^bcde^ ± 1.24 | 588.224^*^ | =<0.001^*^ |
| **% of inhibition** |  |  | **4.54** | **48.25** | **41.98** |  |  |
| **p_control_** |  | <0.001^*^ | <0.001^*^ | <0.001^*^ | <0.001^*^ |  |  |
| **p_0_** |  |  | 0.034^*^ | <0.001^*^ | <0.001^*^ |  |  |
|  |  |  | p_1_<0.001^*^,p_2_<0.001^*^,p_3_=0.004^*^ | | |  |  |
| **20%**  **% of proliferation** | 100 ± 0 | 107.5^ade^ ± 2.86 | 97.53^a^ ± 1.47 | 81.23^acde^ ± 1.18 | 80.10^ae^ ± 3.05 | 104.055^*^ | <0.001^*^ |
| **% of inhibition** |  |  | **9.27** | **24.44** | **25.49** |  |  |
| **p_control_** |  | 0.008^*^ | 0.600 | <0.001^*^ | <0.001^*^ |  |  |
| **p_0_** |  |  | 0.001^*^ | <0.001^*^ | <0.001^*^ |  |  |
|  |  |  | p_1_<0.001^*^,p_2_<0.001^*^,p_3_=0.958 | | |  |  |
| **10%**  **% of proliferation** | 100 ± 0 | 103.2^a^ ± 3.25 | 95.09^a^ ± 2.07 | 88.13^abe^ ± 2.49 | 81.43^a^ ± 1.99 | 46.814^*^ | <0.001^*^ |
| **% of inhibition** |  |  | **7.86** | **14.60** | **21.09** |  |  |
| **p_control_** |  | 0.458 | 0.126 | 0.001^*^ | <0.001^*^ |  |  |
| **p_0_** |  |  | 0.009^*^ | <0.001^*^ | <0.001^*^ |  |  |
|  |  |  | p_1_=0.023^*^,p_2_<0.001^*^,p_3_=0.028^*^ | | |  |  |
| **5%**  **% of proliferation** | 100 ± 0 | 101^ab^ ± 0.45 | 99.14^a^ ± 1.98 | 92.77^ab^ ± 1.96 | 84.06^a^ ± 1.02 | 84.240^*^ | <0.001^*^ |
| **% of inhibition** |  |  | **1.84** | **8.15** | **16.77** |  |  |
| **p_control_** |  | 0.897 | 0.928 | <0.001^*^ | <0.001^*^ |  |  |
| **p_0_** |  |  | 0.849 | <0.001^*^ | <0.001^*^ |  |  |
|  |  |  | p_1_=0.001^*^,p_2_<0.001^*^,p_3_<0.001^*^ | | |  |  |
| **2.5%**  **% of proliferation** | 100 ± 0 | 100.3^ab^ ± 1.28 | 99.62^a^ ± 1.12 | 96.47^abc^ ± 2.87 | 86.30^ab^ ± 1.13 | 42.380^*^ | <0.001^*^ |
| **% of inhibition** |  |  | **0.68** | **3.82** | **13.96** |  |  |
| **p_control_** |  | 0.999 | 0.998 | 0.116 | <0.001^*^ |  |  |
| **p_0_** |  |  | 0.982 | 0.081 | <0.001^*^ |  |  |
|  |  |  | p_1_=0.179,p_2_<0.001^*^,p_3_<0.001^*^ | | |  |  |
| **F** | – | 36.918^*^ | 59.857^*^ | 101.904^*^ | 36.827^*^ |  |  |
| **p_4_** | – | <0.001^*^ | <0.001^*^ | <0.001^*^ | <0.001^*^ |  |  |

Data was expressed using Mean ± Standard deviation (SD)

F: F for One-way ANOVA test, Pairwise comparison between each two groups was done using Post Hoc Test (Tukey)

p: p value for comparing between the studied groups

P_control_: p value for comparing between the control and each other groups

p_0_: p value for comparing between the sera negative for parasitic IgG and other groups

p_1_: p value for comparing between the *S.mansoni* IgG^+^sera and *T.spiralis* IgG^+^sera

p_2_: p value for comparing between the *S.mansoni* IgG^+^sera and *T. gondii* IgG^+^sera

p_3_: p value for comparing between the *T.spiralis* IgG^+^sera and *T. gondii* IgG^+^sera

p_4_: p value for Student t-test for comparing between the different studied concentrations of the same IgG-positive sera

*: Statistically significant at p ≤ 0.05

a: Significant with **40%**

b: Significant with **20%**

c: Significant with **10%**

d: Significant with **5%**

e: Significant with **2.5%**

**Table (5): Comparison of maximal inhibitory percentages of different parasitic antigens and their corresponding parasitic IgG-positive sera on HT-29 colorectal cells and HepG2 hepatocellular carcinoma cells**.

| **Cancer cell lines** | **Concentration** | ***S. mansoni*** | ***T. spiralis*** | ***T. gondii*** | **F** | **p** |
| --- | --- | --- | --- | --- | --- | --- |
| **% of HT-29 Colorectal Cancer**  **cell Inhibition** | **Parasitic antigens**  **25 µg/ml** | 31.72 ± 0.07 | 36.65 ± 0.29 | 31.23 ± 0.66 | 153.178^*^ | <0.001^*^ |
|  |  | p_1_<0.001^*^,p_2_=0.377,p_3_<0.001^*^ | | |  |  |
|  | **Sera positive**  **for parasitic IgG 40%** | 37.17 ± 0.13 | 49.90 ± 0.89 | 50.43 ± 1.90 | 115.112^*^ | <0.001^*^ |
|  |  | p_1_<0.001^*^,p_2_<0.001^*^,p_3_=0.855 | | |  |  |
|  | **t** | 63.513^*^ | 24.492^*^ | 16.564^*^ |  |  |
|  | **p_4_** | <0.001^*^ | <0.001^*^ | <0.001^*^ |  |  |
| **% of HepG2 hepatocellular carcinoma Inhibition** | **Parasitic antigens**  **100 µg/ml** | 8.03 ± 2.35 | 38.27 ± 1.96 | 34.68 ± 3.07 | 130.896^*^ | <0.001^*^ |
|  |  | p_1_<0.001^*^,p_2_<0.001^*^,p_3_=0.261 | | |  |  |
|  | **Sera positive**  **for parasitic IgG**  **40%** | 4.54 ± 1.40 | 48.25 ± 2.30 | 41.98 ± 1.03 | 604.845^*^ | <0.001^*^ |
|  |  | p_1_<0.001^*^,p_2_<0.001^*^,p_3_=0.009^*^ | | |  |  |
|  | **t** | 2.211 | 5.731^*^ | 3.904^*^ |  |  |
|  | **p_4_** | 0.091 | 0.005^*^ | 0.017^*^ |  |  |

Data was expressed using Mean ± Standard deviation (SD)

F: F for One-way ANOVA test, Pairwise comparison between each two groups was done using Post Hoc Test (Tukey)

p: p value for comparing between the studied groups

p_1_: p value for comparing between *S. mansoni* and *T. spiralis*

p_2_: p value for comparing between the *S. mansoni* and *T. gondii*

p_3_: p value for comparing between the *T. spiralis* and *T. gondii*

p_4_: p value for comparing between inhibitory percentages of parasitic antigens and their corresponding parasitic IgG-positive sera

*: Statistically significant at p ≤ 0.05
